# Untangling Brain-Wide Dynamics in Consciousness by Cross-Embedding

**DOI:** 10.1371/journal.pcbi.1004537

**Published:** 2015-11-19

**Authors:** Satohiro Tajima, Toru Yanagawa, Naotaka Fujii, Taro Toyoizumi

**Affiliations:** 1 RIKEN Brain Science Institute, Hirosawa, Wako, Saitama, Japan; 2 Department of Neuroscience, University of Geneva, CMU, Genève, Switzerland; 3 Department of Computational Intelligence and Systems Science, Tokyo Institute of Technology, Midori-ku, Yokohama, Kanagawa, Japan; University of Toronto, CANADA

## Abstract

Brain-wide interactions generating complex neural dynamics are considered crucial for emergent cognitive functions. However, the irreducible nature of nonlinear and high-dimensional dynamical interactions challenges conventional reductionist approaches. We introduce a model-free method, based on embedding theorems in nonlinear state-space reconstruction, that permits a simultaneous characterization of complexity in local dynamics, directed interactions between brain areas, and how the complexity is produced by the interactions. We demonstrate this method in large-scale electrophysiological recordings from awake and anesthetized monkeys. The cross-embedding method captures structured interaction underlying cortex-wide dynamics that may be missed by conventional correlation-based analysis, demonstrating a critical role of time-series analysis in characterizing brain state. The method reveals a consciousness-related hierarchy of cortical areas, where dynamical complexity increases along with cross-area information flow. These findings demonstrate the advantages of the cross-embedding method in deciphering large-scale and heterogeneous neuronal systems, suggesting a crucial contribution by sensory-frontoparietal interactions to the emergence of complex brain dynamics during consciousness.

## Author Summary

Advances in recording technologies have enabled the acquisition of neuronal dynamics data at unprecedented scale and resolution, but the increase in data complexity challenges reductionist model-based approaches. Motivated by generic theorems of dynamical systems, we characterize model-free, nonlinear embedding relationships for wide-field electrophysiological data from behaving monkeys. This approach reveals a universality of inter-areal interactions and complexity in conscious brain dynamics, demonstrating its wide application to deciphering complex neuronal systems.

## Introduction

The brain is a complex system with restless activity dynamics [1]. These dynamics are generated and maintained by interactions among variety of neural populations inside the brain. How brain-wide interaction produces specific dynamics underlying cognitive functions is a central question in neuroscience [2,3]. While recent theories propose that complex dynamics generated by integrative and recurrent interactions among brain areas form a universal foundation of conscious brain state [4], it is unknown how complex dynamics support consciousness and what specific circuits in the brain generate the complex dynamics.

Recent experimental results of high-dimensional recordings highlighted the importance of various different aspects of dynamics in individual cortical areas underlying sensory, motor, or cognitive functions [5–9]. A challenging point in characterizing brain-wide dynamics is that the complexity and diversity, which many reductionist model-based approaches neglect, may be an irreducible feature supporting cognitive processes [4]. A dilemma is that overly realistic and detailed simulations often require a number of unknown parameters and can obscure physiological principles [3,10]. It is, therefore, not straightforward to instantiate an appropriate reductive model that capture dynamical complexity and diversity across multiple brain areas [3]. Another stream of research directly evaluates statistical features in data. Although some approaches evaluate complexity of neural activity (e.g. the correlation dimension [11] or the algorithmic complexity [12]) and others evaluate neural interaction (e.g. the functional connectivity [13] or the Granger causality [14]), there are few methods that relate these two features in a consistent interpretable manner.

Here we introduce an alternative approach that can address how brain-wide nonlinear neural interaction generates complex dynamics, with minimal assumptions. We focus on a generic mathematical property termed *delay-embedding* [15,16] in nonlinear dynamical systems, which enables topological reconstruction of global attractor dynamics based on local temporal sequences. We developed an extended delay-embedding method to provide an accurate estimate of dynamical complexity, i.e., the dimensionality of a reconstructed attracter, under the presence of confounding factors in real data, based on a random projection technique. Relying only on the topological aspect of reconstructed attractor dynamics, the method allows us to shortcut several specific model assumptions and a-priori dimensionality reduction of data, and yet provides a clear explanation for how nonlinear interactions generate complexity of dynamics.

We first illustrate the principle of our approach with simple models (where we know the true underlying dynamical system), showing that it reliably estimates interactions among observed nodes and the dynamical complexities that reflect the interactions, despite variations in signal timescale and noise. Next, we apply the method to wide-field electrophysiological recordings from awake and anesthetized monkey cortex, reporting a novel hierarchical organization of cortical areas as a universal correlate of conscious brain state. In this hierarchy, the dynamical complexity increases along the directed cross-area interaction from the visual to frontoparietal areas. This can reconcile two contrasting views on conscious process—concerning whether conscious processes are localized to specific “workspace” areas [17,18] or distributed across the entire system [4,19,20]—from the viewpoint of information coding by dynamical systems. The high dynamical complexity in the workspace area is accompanied by structured interactions across cortical areas under conscious states. Remarkably, we find that brain-state distinction is reflected in the high-dimensional temporal sequences, but not in snapshots of neural activity, emphasizing the functional importance of dynamics. Finally, we discuss possible physiological mechanisms that account for the current results, proposing a likely (but to date not thoroughly examined) contribution of bottom-up interaction (that originates from sensory cortex) to generate the state-dependent complexity of frontoparietal dynamics.

## Results

### Embedding

**Fig 1:**
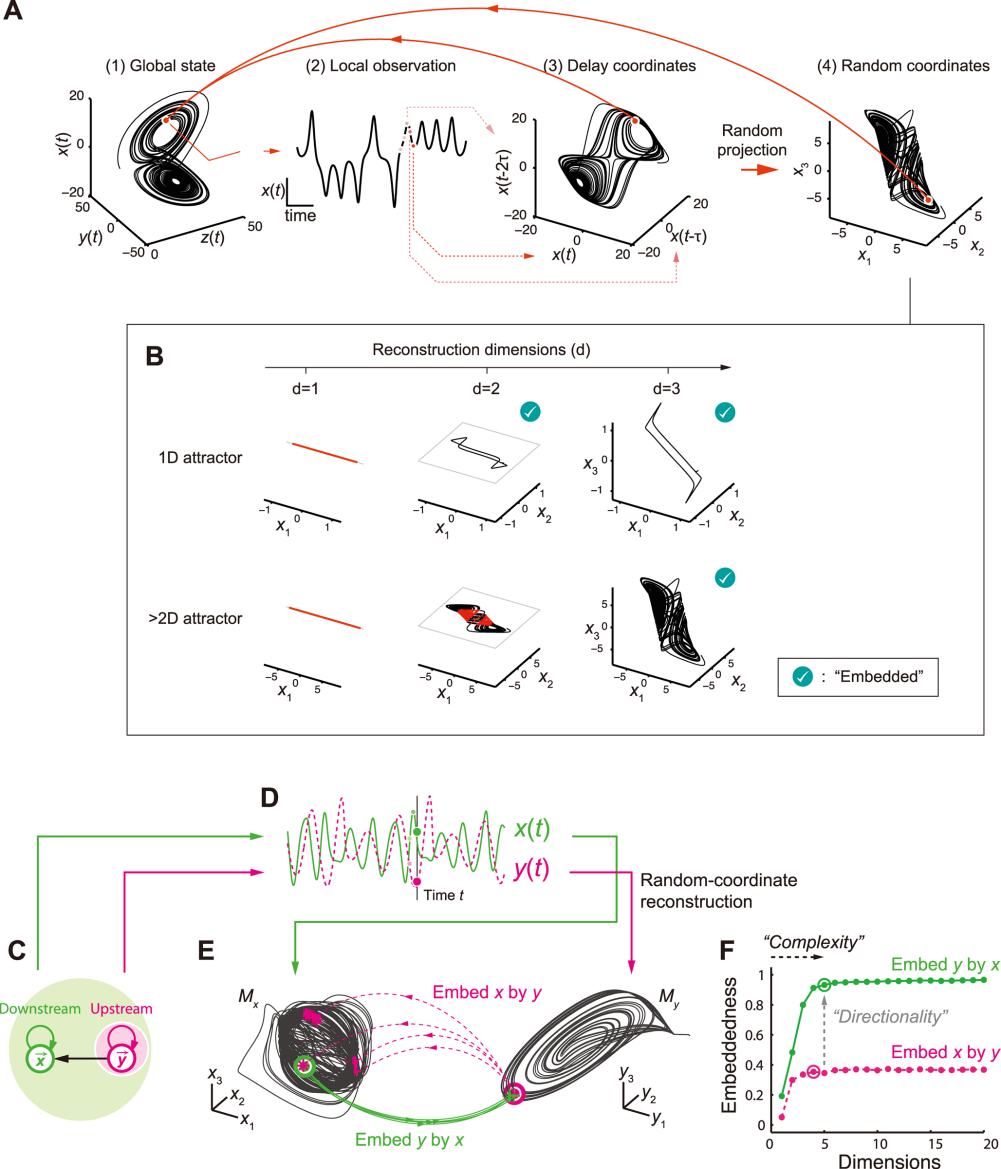
Cross-embedding analysis of complex network dynamics based on state-space reconstruction. (A) Example of randomized delay-coordinate reconstruction assuming we observe dynamics determined with three-dimensional differential equation. (1) Global state is embedded using (2) temporal sequence of single observable of system. Attractor topology in global state space is fully recovered in (3) delay-coordinate of single observable. This relationship is maintained after (4) random linear projection of delay-coordinate space. Thus, there exists unique one-to-one map from (4) to (1), given sufficient number of coordinates. (B) The reconstruction needs sufficient dimensionality. Whether we have embedding or not depends on the relation between the reconstruction dimension (*d*) and the underlying attractor dimension (*d*_*A*_). For example, a two-dimensional delay-coordinate space generically embeds one-dimensional attractor (e.g., a limit cycle, top row), except for a finite number of points. Higher dimensionality is required to embed more complex dynamics (bottom row). Generally, a dimensionality larger than that of an attractor (*d* > *d*_*A*_) is necessary and sufficient for the embedding—to distinguish almost all states of the system [16] although perfect embedding without no self-intersection requires *d* > 2*d*_A_ [15]; the current cross-embedding protocol utilizes the former property. The Figure explores 1, 2 or 3 embedding dimensions after randomly projecting original delay-coordinates. Red regions indicate self-intersection with unignorable volume, reflecting incomplete embedding. (C) Unidirectional interaction between two modeled systems (directionally coupled Rössler systems, see Materials and Methods for equations). Downstream has information for whole system (light green) whereas upstream only has information on itself (light magenta). (D) Example of observed upstream and downstream signals. (E) The cross-embedding based on the random-coordinate state-space reconstructions. *M*_*x*_ and *M*_*y*_ respectively represent the downstream (*x*) and upstream (*y*) attractors based on single observed variable from each system. Colored arrows depict how nearby states in one attractor (disk: 20 nearest-neighbor states) are mapped onto states (dots) in other attractor (green: from x to y and magenta: from y to x). (F) Relationship between coordinate dimensions (number of randomized coordinates used for attractor reconstruction) and embeddedness (how accurately one node’s history can predict another node’s state). The circled dots represent *complexity* estimates, which were determined as minimum embedding dimension that provided ≥ 95% of optimal embeddedness measure (correlation between actual and forecast signals. See Materials and Methods).

**Fig 2:**
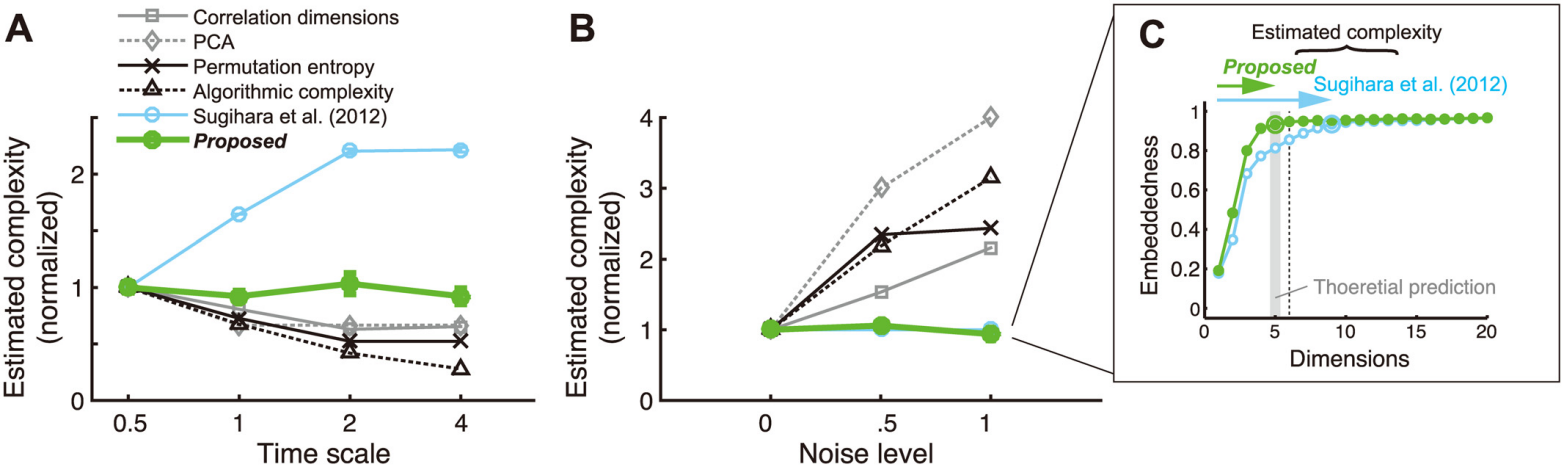
Cross-embedding with random coordinates dissociates interaction-relevant *complexity* from timescale or noise variations. (A) *Complexity* estimates based on embedding *y* with *x* in the system used in Fig 1C–1F, where timescale of signal was varied. The estimates with each method were shown as relative values to those at timescale = 0.5. The results were similar in embedding *x* with *y*. (B) The results where the observation noise level was varied. The noise level corresponds to the standard deviation of the (zero-mean) normal distribution, from which noise were generated. The results are shown as relative values to those at noise level = 0. Although both the standard- and random-coordinate embedding methods (circle symbols) are invariant to noise level in terms of the relative values, the random-coordinate method estimates the true complexity more accurately in absolute values (see panel C). (C) Coordinate randomization avoids overestimation of *complexity*. The markers and error bars indicate the averages ±s.e.m. across 10 trials with different initial states of dynamics. Here, the pre-normalized *complexity* estimates using regular delay coordinates, (*x*_*t*_, *x*_t-τ_ …) (open symbols), are compared with results in Fig 1C (*y* embedded by *x*) which used random delay coordinates (closed symbols); other conventions follows those in Fig 1F. The theoretically predicted embedding dimension of *x* (shaded bar) were accurately estimated using random coordinates, while the previous method using standard delay-coordinates tends to overestimate it, even exceeding the total number of nodes (dotted line) in the simulated system, which upper bounds the theoretically possible attractor dimensions by construction.

**Fig 3:**
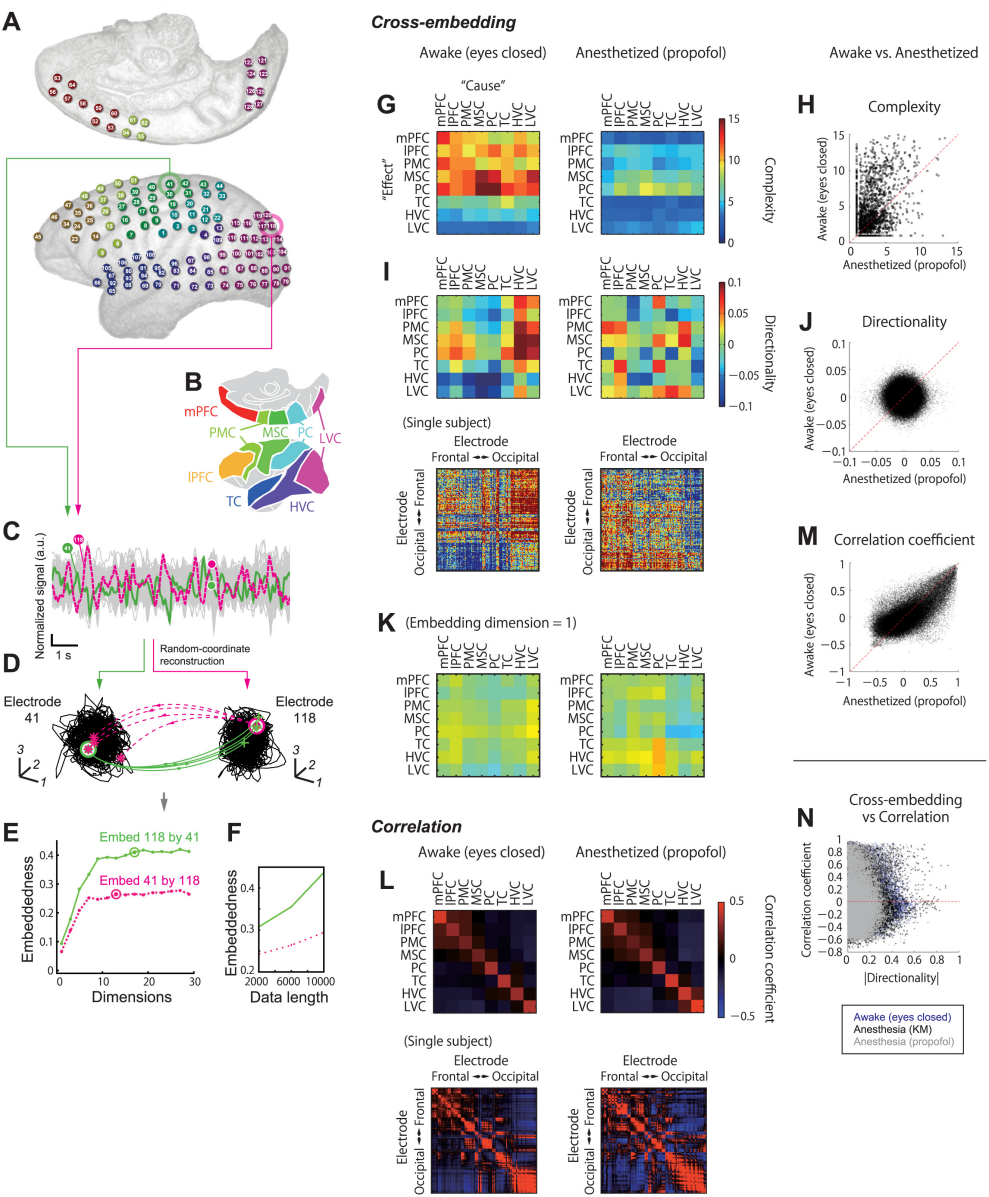
Cross-embedding analysis reveals large-scale cortical interaction, which can be missed by correlation-based statistics. (A) ECoG electrode loci in a representative subject. (B) Illustration of cortical areas covered by the present recording system. (C) Example of ECoG signals. Gray traces represent the signal from the all electrodes superimposed; green and magenta traces show the signals in an example electrode pair (electrodes 41 and 118). (D) Cross-embedding analysis based on the state-space reconstructions for the example electrode pair, corresponding to Fig 1E and 1F. (E) Relationship between coordinate dimensions and embeddedness in the example electrode pair, corresponding to Fig 1F. (F) Optimal embeddedness values as functions of data length (number of data points) used in the analysis. The line colors corresponds to those in panel E. Note that the embeddedness values improve as data length increases, which is a hallmark of causally coupled deterministic dynamics [21]. (G-K) Cross-embedding results. (G) The *complexity* of area-to-area interaction are shown in the matrix formats, which show the results of interaction from areas specifying columns to areas specifying rows. (H) Scatter plot of *complexity* in awake vs. anesthetized conditions; each dot represents a *complexity* value for each “effect” electrode, averaged across “cause” electrodes. Dashed red line shows equality. (I) (Top) The *directionality* of area-to-area interaction are shown in the matrix formats. (Bottom) The *directionality* among individual electrodes in a single subject. (J) Scatter plot of *directionality* in awake vs. anesthetized conditions; each dot represents the values for each electrode pair. (K) The same as panels I, except for that the *directionality* values were computed based on the cross-embedding analysis with limiting the embedding dimension to 1. (L-N) Correlation analysis of snapshot electrode signals. (L) (Top) Average correlation among areas. (Bottom) Correlation among individual electrodes in a single subject. (M) Scatter plots of *complexity*, *directionality*, and correlation coefficient in awake vs. anesthetized conditions; each dot represents the values for each electrode pair. (N) Comparison between cross-embedding-based *directionality* and correlation. Dashed red line shows zero value of correlation. Panels G-L, N and O show the pooled results across the four subjects. LVC: lower visual cortex; HVC: higher visual cortices; TC: temporal cortex; PC: parietal cortex; MSC motor and somatosensory cortex; PMC: premotor cortex; lPFC: lateral prefrontal cortex; mPFC: medial prefrontal cortex

In dynamical systems, causally interacting variables (say, electrode signals *x*, *y* and *z*) share a trajectory in the state space, (*x*, *y*, *z*) [Fig 1A, (1)]—that is, each time point corresponds to a location on the common set of realizing states (“attractor”) in this space. It means that once we specify the time evolution of a variable, the other variables’ behaviors are also constrained via this set (e.g., the dynamics of multiple electrodes can be estimated based on a single electrode’s dynamics). Indeed, mathematical theorems guarantee that the temporal sequence of a single variable [“local observation”, Fig 1A, (2)] has sufficient information about the underlying high-dimensional system dynamics. This is sufficient for complete reconstruction of the original global attractor topology [Fig 1A, (3)]; the global state has one-to-one mapping to a local temporal sequence, or equivalently, a location within the delay-coordinate state-space [Fig 1A, from (3) to (1)]. This property is called “embedding,” a general principle in deterministic dynamical systems.

Based on this property, a protocol for inferring causation in real systems was recently proposed in ecology [21], using nearest-neighbor forecasting techniques [22,23]. It allows us to detect causal interactions and their directionality because a “downstream-node” generally has sufficient information to estimate the dynamics of the “upstream-nodes” but not vice versa (more details are described in the next section). However, this protocol alone does not tell us how the causal interactions shape emergent dynamics, which is the central issue in the present study (and in many other neuroscience studies). In addition, we show that the estimate of dynamical complexity is severely affected by signal timescale, when we reconstruct the state-space based on the standard delay-coordinate (as in the previous work [21], which focused on detecting causality but not on accurately quantifying the attractor complexity). Therefore it is problematic to naïvely apply the original protocol to a highly heterogeneous system such as the brain [e.g., different cortical areas have different timescales of dynamics [24]].

**Fig 4:**
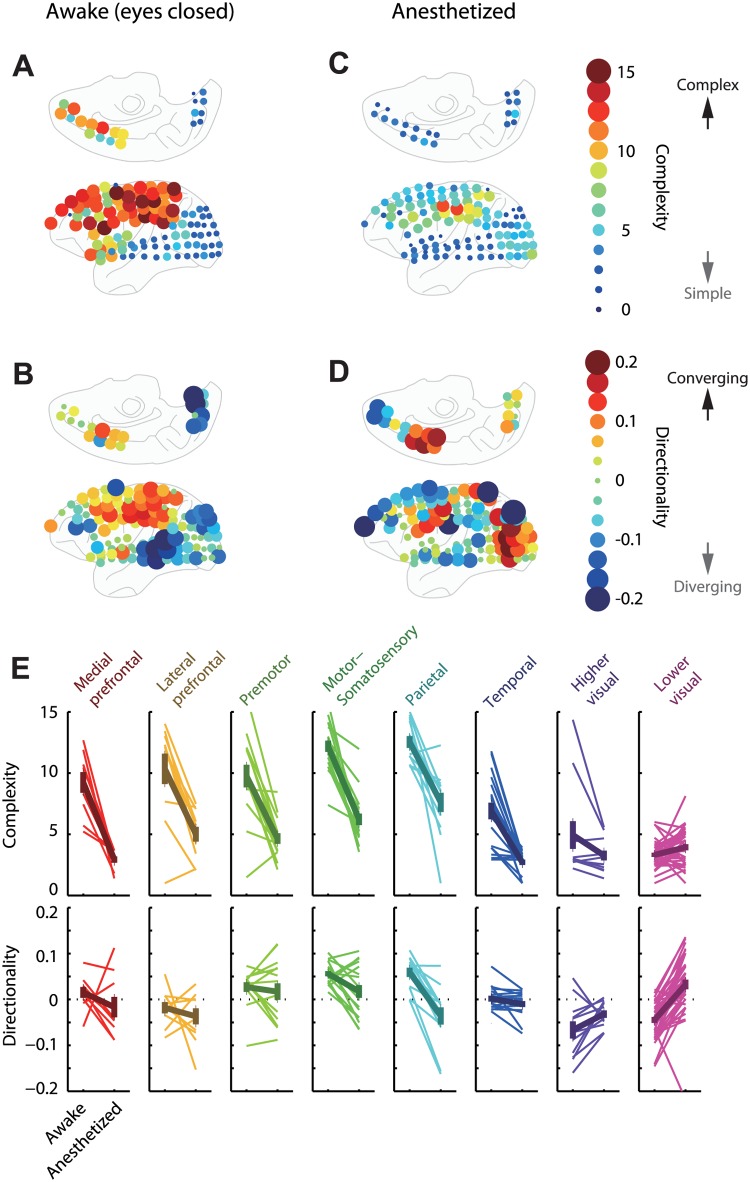
Dynamical complexity and directed interaction within awake and anesthetized brain. Unlike anesthetized brain, awake brain exhibited clear differentiation between frontoparietal and occipital areas. This figure provides results in a single representative experiment, where subject state was changed from awake (A and B), eyes-closed condition to anesthesia by injection of propofol (C and D). Loci of bubbles correspond to electrode positions; the colors of bubbles indicate (A and C) the *complexity* or (B and D) the *directionality* averaged across forecast electrodes. (The absolute values of *complexity*/*directionality* are also indicated by sizes of bubbles, for visibility.) (E) Area-wise *complexity* and *directionality*. Thin lines correspond to the individual electrodes, and thick lines indicate averages and standard errors across electrodes within each area.

We extended the previous approach to link the causal interaction and the dynamical complexity via an embedding-based relationship between time-series variables (hereafter called *cross-embedding*). The key idea is to utilize the fact that the embedding is invariant to the coordinate transformation—instead of the standard delay coordinates, here we use a random projection of them to reconstruct the attractor dynamics [Fig 1A, (4)]. It is also intuitively understandable that virtually any random projection does not break the topology of the attractor (except for special cases), which is a consequence of random projection theory [25]. A critical requirement for embedding is that the dimensions, *d*, of the state-space for reconstruction have to be greater than the attractor’s dimension, *d*_*A*_ (Fig 1B; see the figure legend for additional comments on the relationship between *d* and *d*_*A*_). We make use of this necessity condition for quantifying the effective attractor dimensionality in local dynamics, which is relevant to interaction from other areas. Before moving on to real data analysis, the following two sections provide simplified examples to describe the general cross-embedding principle and demonstrate how the proposed method dissociates the effective dimensionality from other factors such as the timescale of dynamics.

### Cross-embedding reveals interaction-relevant complexity of dynamics: “*downstream complexity”*

The major innovation of our cross-embedding framework is in the simultaneous reconstruction of directionality in interaction (simply denoted by *directionality* hereafter) and a new dynamical complexity measure (denoted by *complexity*). This is achieved by extending the causality estimation protocol by Sugihara et al. [21] to the dynamical dimensionality domain. It leads to a core theoretical prediction that we refer to as *downstream complexity*: “dynamical complexity increases along directed interaction.” Note that we mention the “downstream” in terms of functional network interaction, not directly associated with the biological or anatomical definition of “downstream areas.”

To have an intuition about this, let us consider a simple model example (Fig 1C), where two recurrent systems (e.g., brain areas *x* and *y*) interact in an asymmetric manner (*y* affects *x* but not vice versa). The aim is to identify their interaction based on two observed time series [*x*(*t*) and *y*(*t*)] from each system (Fig 1D) (which corresponds to inferring interaction among cortical areas based on the electrode signals recorded from them). Since the interaction is only from *y* to *x*, the history of *x* has information about *y*, but not vice versa. We call it “*x* embeds *y*” in this paper. [More formally, we have an one-to-one mapping from attractor manifold *M*_*x*_ to *M*_*y*_, which are respectively reconstructed from the time series *x*(*t*) and *y*(*t*) as random projections of the delay-coordinate reconstructions.] This generally happens if and only if *y* causally influences *x*; it means, conversely, we have no one-to-one mapping from *M*_*y*_ to *M*_*x*_ (Fig 1E). It is a direct consequence of a mathematical theorem proven by Stark [26]. Furthermore, armed with this mathematical insight, it is predicted that the downstream attractor, *M*_*x*_, is generically more “complex” than the upstream one, *M*_*y*_. This is because, as *x* is perturbed by *y*, *x*’s dynamics live in a state space not of *x* alone, but of (*x*, *y*), which generally has higher dimensionality than *y* alone. Despite the fact that we only assume the validity of delay embedding here, we have a strong theoretical prediction: asymmetry in causal interaction should be accompanied by a hierarchy of dynamical complexities, which we directly and quantitatively test using electrophysiological recordings from monkeys, as described later.

For quantitative assessments of the cross-embedding relationships, we introduced two measures summarizing the cross-embedding relationships: *directionality* and *complexity* (the full details of the analyses are described in Materials and Methods). First, we defined the *directionality* of interaction from *y* to *x* by the difference in embeddedness of *y* by *x* minus that of *x* by *y* (Fig 1F). Here the embeddedness between the variables is quantified, essentially by seeing whether the time indices of nearby data points on attractor *M*_*x*_ are also clustered on *M*_*y*_ (Fig 1E; see also Materials and Methods). This embeddedness takes a value in between 0 and 1. Given a sufficient data length, a positive value of *directionality* from *y* to *x* indicates an asymmetric causal interaction directed to *x* [21]. Next, we introduced a new measure, *complexity*, of interaction from *y* to *x* by the required dimensions of *M*_*x*_ to identify the corresponding locus on *M*_*y*_. The embeddedness is given as a function of reconstructing dimensions and therefore the *complexity* was defined as the dimensions at which the embeddedness is saturated (Fig 1F; Materials and Methods). Coordinate randomization is crucial to avoiding systematic errors in *complexity* that might arise due to a finite data size (see next section). All the analyses are based on pairwise node-to-node relationships, and thus computations are scalable to high-dimensional networks.

As the previous study [21] did, we allow the data to include some noise, although the embedding theorem in the mathematically rigorous sense assumes deterministic dynamical systems. In the practical data analysis below, we do not assume that the data are noise-free, but expect that the data contain some noticeable components that reflect underlying deterministic dynamics. If we could reconstruct the attractor dynamics by the randomized delay-coordinate state space, even under some approximations, we are able to estimate the directional information flow and dynamical complexity. Note that incomplete recovery of upstream dynamics does not necessarily indicate a breakdown of our deterministic assumption; the present cross-embedding method is generally data-expensive because required data length exponentially increases with the dimension of a downstream attractor. Nevertheless, it turns out that our method can still extract meaningful features of an underlying dynamical system even with finite data size and in the presence of small noise, as shown in the next section (see also S4 Fig).

### Robustness under divergent timescales and variability

In realistic setups that include observation noise and limited data points with various length of signal autocorrelation (or “timescale”), quantifying the attractor complexity is not necessarily straightforward, as already pointed out in dynamical systems studies [27,28]. Therefore, we examined the robustness of our *complexity* measure against variation in signal timescales and the level of observation noise. If the estimate is systematically affected by those signal properties, it complicates the interpretation of the complexity measure because different brain areas are known to exhibit different timescales [24] and experimental observations inevitably involve noise. We compared the present *complexity* measure to the five other related measures (Materials and Methods): (i) correlation dimension [29], (ii) dimensionality derived from principle component analysis (PCA), (iii) permutation entropy [30,31], (iv) algorithmic (Kolmogorov-Chaitin) complexity [32,33], and (v) the optimal embedding dimension using the standard (non-randomized) delay-coordinate [using the algorithm by [21], which was not specifically designed to measure the complexity]. The measures (i) and (ii) were applied to the attractor manifold that was reconstructed within the random delay-coordinate for each electrode signal.

Using the simulated dynamics with an artificial system (Materials and Methods), we confirmed two points. First, the present complexity measure is almost orthogonal to the timescale (Fig 2A) and observation noise (Fig 2B). We also confirmed that the randomization of the coordinates worked for avoiding the overestimation of complexity for relatively slow dynamics (Fig 2C). Second, the conventional complexity measures can be sensitive to the signal properties such as timescale or observation noise. In particular, while the measures (i)-(iv) have been widely used in neuroscience [11,12,34–39], we need to be careful with their interpretation in the presence of such confounding factors. In contrast, our new measure is advantageous for fair comparison of dynamical complexity across heterogeneous brain dynamics. Together, our new measure based on cross-embedding has advantages in providing a reliable measure of dynamical complexity under potential heterogeneity in timescale or observation noise. Note that the primary aim of introducing coordinate randomization is the accurate and robust estimation of *complexity*, but not of *directionality*. Directionality of interactions can be estimated accurately without using coordinate randomization if we select the embedding dimensions appropriately. We also confirmed the robust estimation of *complexity* and *directionality* under the variation in system noise (S4 Fig).

### Cross-embedding analysis of large-scale cortical dynamics

Based on the cross-embedding framework, we explored the structure of physiological large-scale neural dynamics that concern distinct brain states. We recorded the large-scale brain dynamics from monkey electrocorticography (ECoG) recordings from most of the hemispheric cortical surface (Fig 3A and 3B).

We performed the aforementioned cross-embedding analysis on the resting-state ECoG signals (Fig 3C–3F), as well as comparing conscious and unconscious brain states. The *complexity* and the *directionality* were first computed for each electrode pair (128×128 = 16384 pairs in total). Although the embeddedness hardly reaches the theoretical maximum value of one (1) with real data under finite recording time, the asymmetry of embedding relation between an electrodes pair was often clear and statistically significant (Fig 3E and 3F). To characterize inter-areal interactions, we then averaged all the *complexity* and *directionality* observed within each set of areas. Notably, the *complexity* varied depending on the targets, but not on the source areas of directed interactions (Fig 3G); this is consistent with the feature that the current *complexity* measure converges to the attractor dimensions of the target with sufficient data (see Materials and Methods). Overall, the awake state showed higher *complexity* than the anesthetized state (Fig 3H; p<10^−5^, sign test, paired samples). On the other hand, the *directionality* depended both on source and target areas (Fig 3I). Although the lower and higher visual cortices tended to show similar properties under the awake condition (Fig 3I, left), no clear structure was observed in the anesthetized state (Fig 3I, right). The structure of *directionality* changed drastically between the awake and the anesthetized conditions, which was further confirmed by the small correlation between these values between the two conditions (Fig 3J; ρ = 0.01, p<10^−4^, Spearman rank correlation test). We also found that the structure of *directionality* was less clear when we used an insufficient number of reconstruction dimensions (e.g., *d* = 1, Fig 3K), indicating the importance of dynamical history, not just momentary activity levels.

Remarkably, the present cross-embedding analysis extracts a correlate of the brain-state change much more clearly than the conventional characterization based on correlation. For example, in terms of correlation among momentary electrode signals, the awake and anesthetized states had similar network structures (Fig 3L), but with weaker correlations in the awake state (p<10^−5^, sign test, paired samples with absolute correlation coefficients). This was confirmed by pair-wise correlation coefficients correlated between the awake and anesthetized states (Fig 3M; **ρ** = 0.18, p<10^−5^, Spearman rank correlation test); the correlation between the two conditions was significant even if the analysis was restricted to electrode pairs straddling different cortical areas to remove trivial distance dependency (ρ = 0.52, p<10^−5^, Spearman rank correlation test; S5A Fig). These results are in contrast to the drastic structural change in the cross-embedding-based *directionality* values between the two conditions (Fig 3I and 3J). Difference in the dynamics was also not obvious when the dimensionality of the multi-electrode signal was reduced by PCA (S2 Fig). Furthermore, we found that electrode pairs showing only weak correlation (correlation coefficient ≈ 0) can have strong asymmetric interaction in terms of *directionality* values (Fig 3N); in both awake and anesthetized conditions, the absolute values of correlation coefficients, and *directionality* for individual electrode pairs, were overall not strongly correlated (-0.0394<ρ<-0.0307, 95% confidence interval of Spearman rank correlation) although they showed weak negative correlation within the three behavioral conditions (Awake-eyes-closed, ρ = -0.02, p<10^−29^; Anesthesia (ketamine-medetomidine), ρ = -0.04, P<10^−46^; Anesthesia (propofol), ρ = -0.02, P<10^−4^; Spearman rank correlation test). These results demonstrate that cross-embedding and correlation extract different aspects of dynamics. Specifically, brain-state dependent cross-areal interaction detected by the *directionality* was not reflected in conventional correlation-based statistics. This fact may explain why some studies [37,40–42] but not others [13,43] have reported changes in the resting-state functional connectivity depending on arousal level.

### Complexity increases along directed interaction in the awake cortex

To relate the cross-embedding properties to the electrode loci on the cortex, we averaged the *complexity* and *directionality* values over all source areas (Fig 4). It demonstrated three major findings: First, dynamical *complexity* was much higher in frontoparietal areas than occipital areas in the awake state (Fig 4A). Second, we found the information *directionality* in the awake cortex from occipital to frontoparietal areas, indicating that latter areas were downstream of the functional network (Fig 4B). This covariation of the *directionality* and *complexity* measures is consistent with the prediction of the embedding theorem that receiving additional information via directed interaction increases dynamical complexity. Third, these characteristic structures of *directionality* and *complexity* seen in the awake cortex disappeared under anesthesia (Fig 4C and 4D). In particular, the *complexity* in the frontoparietal cortex was drastically reduced, resulting in a more uniform distribution of *complexity* across cortical regions (Fig 4C). The electrodes within the same area generally shared these trends (Fig 4E). To summarize, the distinct brain states seemed to be selectively characterized by the *downstream complexity* (the higher dynamical complexity downstream of directed interaction) of a whole-brain functional network.

### Increased downstream complexity is a universal property of conscious neuronal dynamics

**Fig 5:**
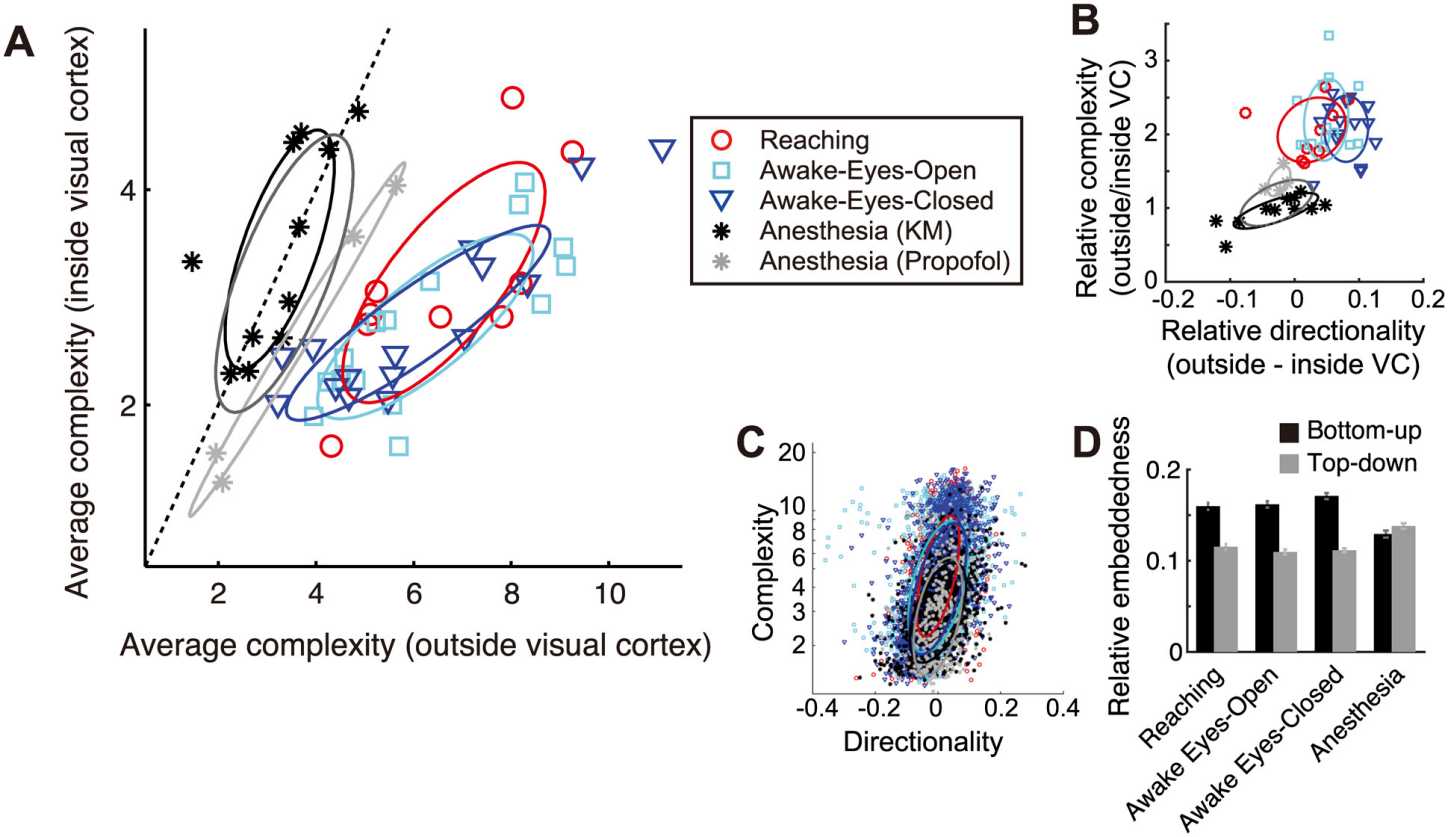
Universality of complex dynamics across different awake conditions. (A) Conscious and unconscious states are differentiated in the space of *complexity* averaged across all areas inside and outside the visual cortices. Markers represent individual experiments. Results from four monkeys have been superimposed. Note that the data from Reaching, Awake-Eyes-Open, and Awake-Eyes-Closed conditions are overlapped. (B) In conscious states, the *complexity* and *directionality* simultaneously increase in the outside visual cortex relative to the inside visual cortex. The convention follows that of panel A. (C) *Complexity* and *directionality* are correlated even in anesthetized condition. Each marker shows averaged *complexity* and *directionality* values (as in Fig 4) in each single electrode. (D) Anesthetization decreases the bottom-up interaction. The relative embeddedness reflects the dynamical coupling after the baseline change correlation is subtracted (Materials and Methods). The black and gray bars indicate the strength of bottom-up (from visual areas to other areas) and top-down (form other areas to visual areas), respectively. In panels A–C, the ellipses indicate the data covariance within individual conditions; the darker gray ellipse indicates the covariance for the data pooling the ketamine-medetomidine- and propofol-induced anesthesia. The dotted line indicates equality of average complexity. VC: visual cortex.

Next, we questioned how universally these dynamical properties were observed across experimental setups and conditions. For example, the *complexity* of dynamics could reflect only the conscious-unconscious difference (i.e., the *level* of consciousness) in the brain state, or could be affected by other cognitive experiences, such as visual stimulus or body movement (i.e., the *contents* of consciousness) [44]. To dissociate these possibilities, we compared the current results with two additional experimental conditions: “Awake-eyes-open” (where monkeys freely viewed their environment, but their body movements were restricted) and “Food-reaching conditions” (where monkeys were seeing and grabbing the food by moving their arms, Materials and Methods). We found that the difference in *complexity* between inside and outside visual cortex distinguished the awake from the anesthetized brain state across individual animals, regardless of substantial differences in behavioral or sensory experiences (Fig 5A).

**Fig 6:**
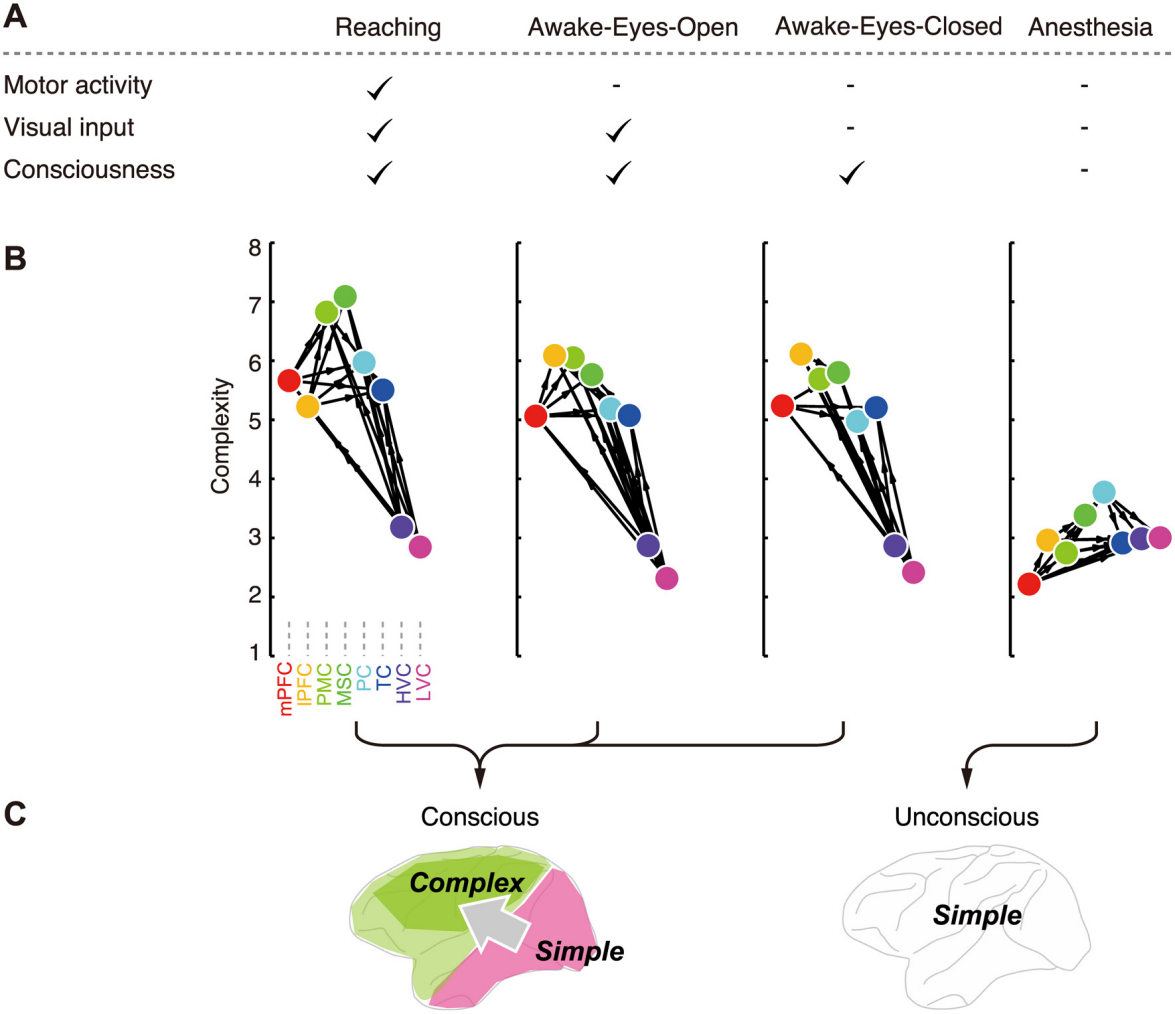
Increased complexity at the cortical downstream differentiates conscious form unconscious brain states. (A) Summary of task conditions. (B) Cortical hierarchy defined by complexity. Areas aligned by *complexity* and relative directed interaction between them have been visualized. Arrows indicate relative information flows from one area to another, which were quantified by average *directionality* for interactions between two areas (only strong information flows exceeding 0.01 are shown). Complexity in visual areas was smaller than that in other areas during awake conditions (reaching: P<0.004; awake-eyes-open and awake-eyes-closed: P<0.00007; sign test, matched samples), and this inter-area difference in complexity was reduced by anesthesia (P<3×10^−8^; Wilcoxon rank sum test, independent samples). (C) Schematic summary of the main findings.

These results suggest that high *complexity* in those areas reflects the conscious brain state rather than specific cognitive processes induced during visual or motor experiences (cf. table in Fig 6A). Notably, high *complexity* was consistently accompanied by downstream areas with high *directionality* across the conditions (Fig 5B). The increase in *complexity* in conscious conditions was significant not within the visual cortex (i.e., the upstream; p>0.2, Wilcoxon rank sum test), but only outside of the visual cortex (i.e., the downstream; p<10^−5^, Wilcoxon rank sum test).

Moreover, further analysis revealed that the *complexity* and *directionality* were significantly correlated across the electrodes in all conditions (Fig 5C; 0.49<ρ<0.62, p<10^−86^, Spearman rank correlation test). This suggests that the co-occurrence of positive *directionality* (network downstream) and high *complexity* is a fundamental property shared between conscious and unconscious states, while the range of *complexity* and spatial structure of interactions differ between these brain states. As we mentioned earlier, this relationship between the complexity and network directionality, although not reported in the previous neuroscientific studies (to our best knowledge), agrees with the theory of directionally-coupled deterministic dynamical systems (as shown in Fig 1C), where the downstream system must have more complex dynamics than the upstream system. Additional analysis supported this view by revealing that the reduction in the top-down interaction (from outside to inside visual cortex) dominates the increase in bottom-up interaction (from inside to outside visual cortex) during the change from the anesthetized to awake-eye-closed condition (Fig 5D; p<10^−5^, sign test, paired samples). To summarize, we found (i) that an increased complexity downstream is generally observed in neural dynamics (regardless of brain state), and (ii) that this property emerges across the global cortical network under conscious brain states, which is likely to be a result of enhanced bottom-up interaction (we further examine this point in a later section).

It was also confirmed that details and operations of the analyses (spatial differentiation, denoising, and orthogonality of the coordinate) did not change the results (S1A–S1D Fig). Finally, the high *complexity* observed in the network downstream is not an artifact of unreliable estimation of *complexity* for weakly interacting nodes because the resulting *complexity* structure was qualitatively and quantitatively unaffected when we restricted our analysis to the strongest interacting nodes (S1E Fig). The results were cross-validated, thus random variability in data does not explain our results. Together, these results revealed a robust structure of the large-scale cortical hierarchy under consciousness, where the visual cortex was located upstream exhibiting simple dynamics, whereas the frontoparietal cortex was located downstream exhibiting complex dynamics (Fig 6B and 6C). Therefore, we conclude that the *downstream complexity* revealed by the present cross-embedding analysis reflects a universal property of neuronal dynamics that can dissociate the conscious and unconscious states.

### Not timescale, but complex temporal pattern is critical for the brain-state dependent hierarchy

In previous studies, the distinct brain states have often been described in terms of their typical timescales of neural dynamics: e.g., slow oscillations in anesthesia/sleep vs. high-frequency fluctuation in wakefulness [45–47]. The present findings on the hierarchy and brain-state dependency of *complexity* are in contrast to the characterization based on timescale because our *complexity* measure was designed to be invariant to the timescale.

**Fig 7:**
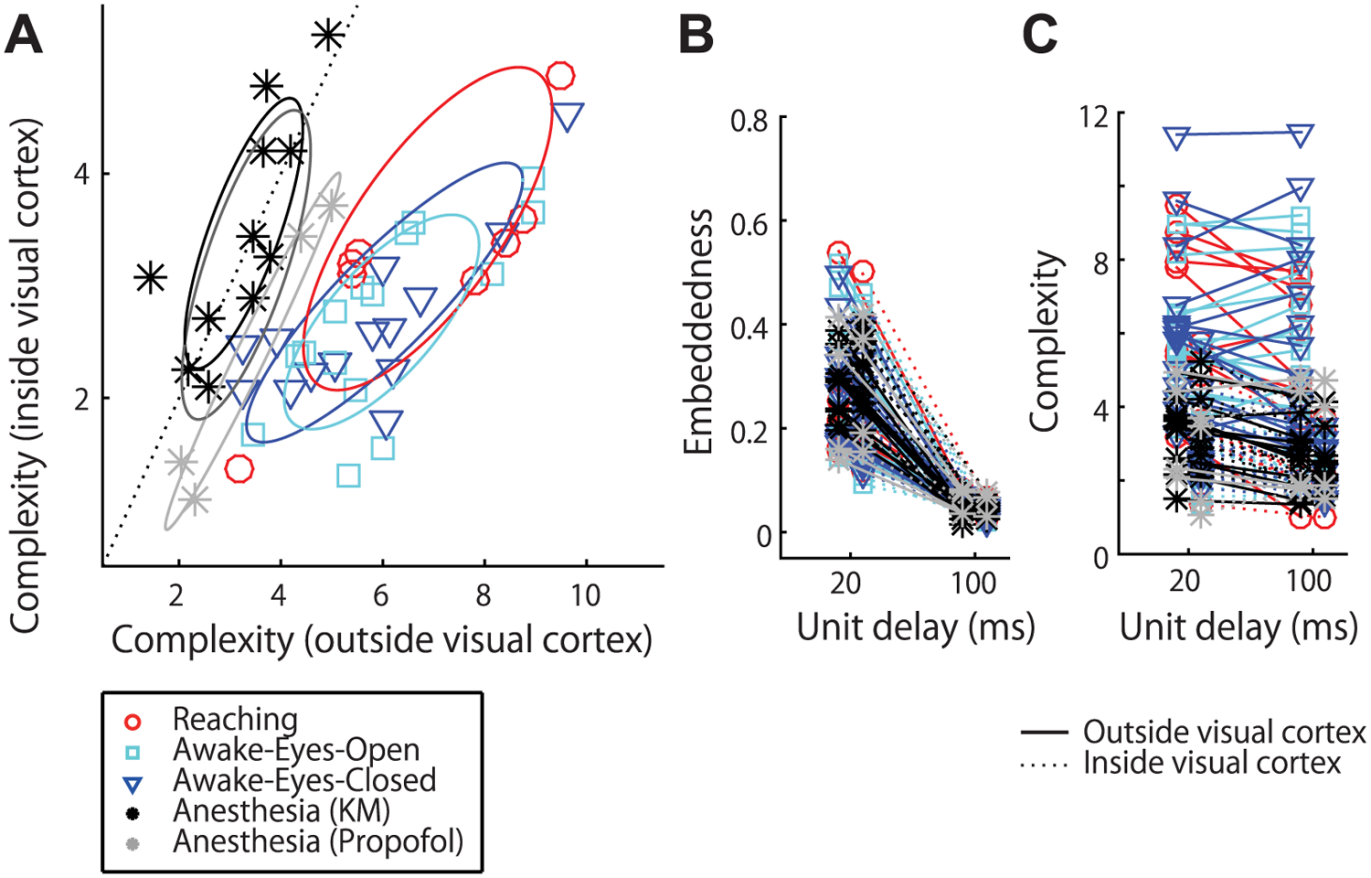
Robustness to variations in timescale. The main result is not affected by lengthening the unit delay although forecasting accuracy (*embeddedness*) was generally degraded by using suboptimal unit delay size. (A) The replications of the results shown in Fig 5A, respectively, where a longer unit delay (100 ms) was used for the state space reconstruction. (B) Optimal *embeddedness* in the all electrode pairs decreased by 0.21 times with the longer unit delay (p<10^−5^, sign test, paired sample across all electrode pairs). The figure shows the embeddedness averaged inside (dashed lines) or outside (solid lines) visual cortex. (C) *Complexity* (averaged inside or outside visual cortex) showed only small or not significant dependency to the unit delay variation (inside visual cortex: changed by 0.90 times, p <10^−4^; outside visual cortex: by 0.80 times, p = 0.5; sign test, paired sample, across all experimental sessions). This robustness validates that *complexity* reflects dimensions of dynamical attractor rather than variations in timescale of signals. In panels D-F, the color code follows that of panel B.

Although its timescale-invariance was confirmed with the simulation (Fig 2A), one might still consider the possibility that the change in *complexity* reflects the difference in the timescale of neural dynamics. To directly exclude this possibility, we replicated the analyses using a different size of the unit delay in the state-space reconstruction. If the *complexity* depended on timescale, this manipulation should yield different results. However, we found that the main results, including the spatial distributions of *complexity* and *directionality*, were robust to the substantial change in the unit delay size (Fig 7A). It should be noted that the use of the different unit-delay can affect the embedding performance (Fig 7B) and the absolute value of *directionality* (changed by 0.36 times), possibly due to the limitation in data length [an interpretation of why 20-Hz unit-delay yielded better embedding performance is that signals with moderately high frequency (e.g., >5 Hz) had large contribution to reconstructing the attractor topology under the noise and limitation of data length]. Nevertheless, the average *directionality* from visual to other areas was still larger in the awake than anesthetized conditions (p<10^−7^; Wilcoxon rank sum test), suggesting the qualitative robustness of the present results. In addition, the *complexity* values themselves were quantitatively maintained across the different unit-delay sizes (Fig 7C), which is reasonable since the *complexity* reflects the attractor dimensionality. Although one might speculate that the *complexity* values were robust to the unit delay if they reflected a temporally scale-free property such as ratio of time-constants between source and target, it is unlikely since the data suggest that the *complexity* primarily depended on the target areas, not on the combination of source and target (Fig 3F). Therefore, our results strongly support the view that this emergent hierarchy of *complexity* accurately reflects the hierarchy of attractor dimensions, which is invariant to the timescale of dynamics.

If timescale is not critical, what determines this hierarchy? Our data suggest that it is the dimensionality of an attractor underlying observed temporal sequences. For example, the correlation structure (which was derived from the momentary signal values) was not sensitive to the difference in brain states, as we have seen before in this paper (Fig 3L). This insensitivity is not caused by the difference in analytical procedure between correlation and cross-embedding because the brain state-dependency was not clear even with the cross-embedding analysis when we limited the embedding dimension to one (Fig 3K). Rather, it is critical to take into account the high-dimensional pattern of temporal sequence, not momentary “snapshots.” Altogether, the current results suggest that the brain-state change from the conscious to unconscious condition is reflected in relatively complex dynamics of neural activities, which is not reduced to conventional statistics such as timescale or correlation.

### Models of interaction-induced complexity increase

What causes the increased *complexity* in the awake frontoparietal cortex? As *directionality* from visual to frontoparietal cortex also increased under the awake conditions, a likely mechanism would be that the increased bottom-up interaction caused the complex frontoparietal dynamics. Alternatively, it is possible that increased *complexity* in the frontoparietal cortex caused the change in the estimate of *directionality* in our cross-embedding analysis.

**Fig 8:**
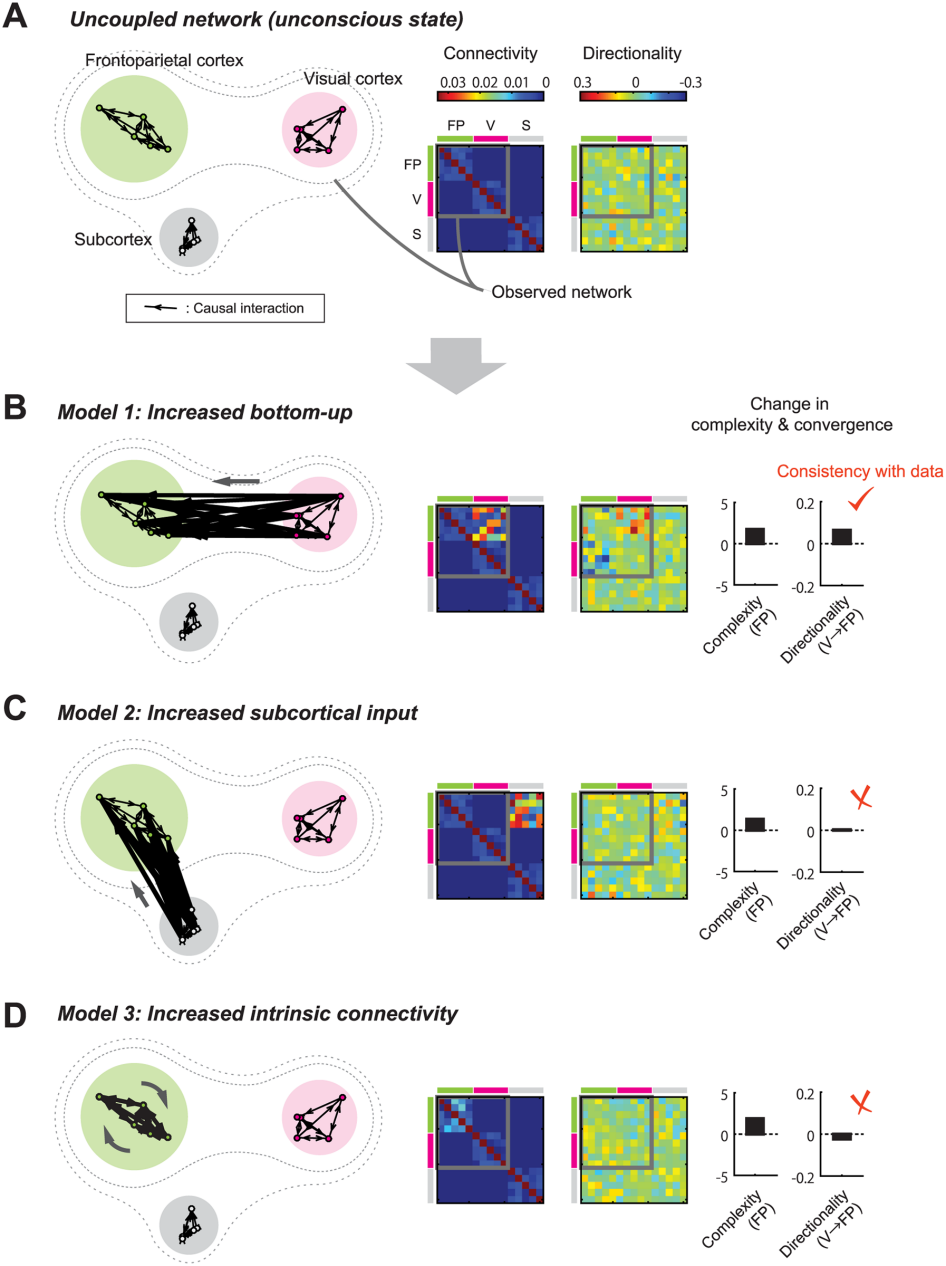
Possible mechanisms of changing frontoparietal dynamics in the conscious brain state. (A) Simplified architecture of nervous system under unconscious (anesthetized) state. In the unconscious state, three clusters (frontoparietal, visual, and subcortical systems) have no strong nonlinear interaction. (B) Model 1: the frontoparietal complexity is increased by the enhanced bottom-up interaction. (C) Model 2: the frontoparietal complexity is increased by subcortical input. (D) Model 3: the frontoparietal complexity is increased by change in the connectivity inside of the frontoparietal system itself. Only Model 1 accounts for the increase in the *directionality* from the visual to the frontoparietal cortex, which was observed in the experimental data. (From left to right columns) Network architecture, mechanistic connectivity matrices, *directionality* matrices derived based on cross-embedding analysis with simulated dynamics, average *complexity* in the frontoparietal cortex, the averaged *directionality* from the visual to the frontoparietal cortex. V: visual cortex. FP: frontoparietal cortex.

To examine this, we explored what mechanism can (or cannot) account for the present results, using a simplified network model (Fig 8). Here, we consider three large clusters of the nervous system: two (visual and frontoparietal cortices) correspond to those observed in the present ECoG experiment while the last one is assumed an unobserved system (e.g., subcortical structure). The contributions of cortical and subcortical mechanisms to the anesthesia-induced loss of consciousness is currently a central subject of debate [48–52]. The unconscious (anesthetized) state was modeled by a network having only weak long-range coupling among the three clusters, but is moderately coupled within individual clusters (Fig 8A), which follows the experimental findings [40]. We simulated individual nodes with a linear-nonlinear model including self-feedback (Materials and Methods). Note that our focus here is not on modeling details of nervous systems but on illustrating the basic mechanisms of how *complexity* and *directionality* change depending on the network properties.

The increase in *complexity* could be caused by three different mechanisms (Fig 8B–8D): (i) increases in the bottom-up interaction from the visual to the frontoparietal cluster, (ii) increased extra-cortical input from the subcortical to the frontoparietal cluster, and (iii) change in intrinsic connectivity within the frontoparietal cluster. Simulation confirmed that all the three mechanisms lead to an increase in *complexity* within the frontoparietal cluster (Fig 8B–8D, fourth column). However, they differ in *directionality* related to the bottom-up interaction from the visual to the frontoparietal cluster (Fig 8B–8D, fifth column). Only the first model (with increased bottom-up interaction) predicted the increase in the visual-to-frontoparietal *directionality*, replicating the present results. In contrast, the other two models showed no increase in this *directionality* measure, which is reasonable because those models do not assume any mechanistic change in terms of the visual-to-frontoparietal interaction. Adding the baseline correlation among nodes did not affect these qualitative results. It should be noted that the results here do not reject partial contributions by (ii) or (iii), or the possibility that the changing *complexity* reflects the variation in indirect interactions between the visual to the frontoparietal areas via subcortical system. Whether direct or indirect, these simulation results suggest that the sensory cortex contributes to shaping the conscious cortical dynamics, posing important constraints on the models of brain-state dependent nervous activities.

## Discussion

We have introduced an embedding-based computational method that bridges cross-area interactions and emergent dynamics with a clear theoretical underpinning, but without making specific or reductive assumptions about brain systems. The method is based only on generic dynamical properties and is readily applicable to systems that are difficult to model. Indeed, it has successfully captured features that can be easily overlooked by conventional reductionist approaches, such as spatiotemporal variations in *complexity* itself (e.g., Fig 4A and 4C). In addition, unlike PCA or power spectrum analyses in temporal frequency domains, this method can fully exploit nonlinear and directed interactions between brain areas. One caveat of the present method is that it may not always detect veritable underlying causal interactions if the data length is restricted. Despite this limitation, the embedding-based characterization of interactions is advantageous over existing prediction-based methods, such as Granger causality or transfer entropy, because it is not confounded by self-predictability [21] (S3B Fig). That is, a node’s own historical trace is generally sufficient to predict its future regardless of the presence or absence of causal interactions from other nodes in a deterministic system.

It is important to discriminate the embedding in a rigorous mathematical sense from that for practical data analysis. Embedding in a rigorous mathematical sense is not defined in the presence of noise. However, in practical applications, embedding-based analysis can extract underlying dynamical features with a resolution limited by the noise. Indeed, the method successfully estimated the dimensionality of an attractor and the structure of interactions in the system (as we showed in S4 Fig, and as demonstrated by Sugihara et al. [21]) unless noise is too large. We believe that the present cross-embedding based analysis yields new insights from the practical viewpoint, in purpose of analyzing deterministic aspects of in neural dynamics. This view is supported by the fact that the present results are fully consistent with a mathematical property of a deterministic system, in which the cortical areas estimated as network downstream have higher *complexity* than the upstream areas. This structure was highly robust to different versions of analytic procedures and parameters (S1 Fig).

Based on this method, we simultaneously characterized the large-scale cortical interaction and the dynamical *complexities* embedded in individual area activities. It revealed that the awake brain has a hierarchical structure of the dynamical *complexity*, where the frontoparietal areas had more complex dynamics than visual areas. Intriguingly, this hierarchy was linked to the directed cross-area interaction from visual to frontoparietal areas. To our best knowledge, this is the first study reporting clear cortical hierarchy in terms of dynamical complexity, as well as its relationship to the global cortical interaction. Moreover, we found that this hierarchy was universal across different behavioral/sensory conditions and disappeared after the loss-of-consciousness induced by either of two different anesthetization methods. These results indicate that this hierarchical structure is correlated with the *level* of consciousness rather than its specific *contents* reflecting perception or action.

The present results demonstrate that the correlation structure was similar (correlated) between awake and anesthetized conditions at a whole-brain scale (Fig 3L and 3M), albeit with slightly larger absolute correlation strength in the anesthetized condition. On the other hand, some previous studies show that the brain states can be differentiated based on correlation-based analysis [53,54]. Although the exact reason behind this discrepancy is beyond the focus of this work, one potential reason is the difference in natures of signals to be analyzed. For example, the correlation based on slow fMRI signal might correspond to a nonlinear sum of the ECoG-based correlation across a wide range of time-delays. We do not exclude the possibility that the correlation-based measure can differentiate the two conditions. Instead, we emphasize that the correlation and *directionality* are conceptually distinct measures—we can intuitively expect that two electrodes can be highly correlated despite week *directionality* in their interaction if the two sites are bidirectionally interacting with the equal strength. Moreover, we observed that the area pairs showing only weak correlation can have large value in the directionality measure, and vice versa (Fig 3O). This result was also robust to the exclusion of intra-areal electrode pairs (S5B Fig).

From the theory and modeling, we proposed that the increasing *complexity* at the frontoparietal cortex under awake conditions is likely to be a consequence of enhanced (direct or indirect) bottom-up interaction from the visual cortex. Another way of accounting for the current data is to assume that the frontoparietal cortex is always downstream of the visual cortex, and that the former simply reflects input from the latter in the unconscious state. In this case, we do not observe any hierarchy among them under unconscious state since the visual and frontoparietal cortices have exactly the same dynamical complexity. When the transition to the conscious state causes some additional input or self-recurrence in the frontoparietal cortex, it no longer simply reflects the visual cortex and can have more complex dynamics. Although theoretically interesting, this hypothesis requires several physiologically questionable assumptions. First, there is currently no evidence supporting the idea that the visual cortex drives the entire cortical dynamic in the unconscious state. Second, it assumes no local recurrent connection within the frontoparietal area under unconsciousness, which is not consistent with the experimental findings (e.g., [40]). Nevertheless, we do not reject such the possibility in this paper. To directly test which mechanism is the most likely, we can stimulate the visual cortex (using transcranial magnetic stimulation or other methods) and compare the efficacy of bottom-up signal transmission from the visual to frontoparietal cortex, among different brain states. If the frontoparietal cortex simply reflects the visual cortex under unconsciousness, the former should precisely reflect the stimulation pattern in the latter. On the other hand, if the bottom-up interaction varies among the distinct brain states (as we hypothesized in the above), the stimulation in the visual cortex would have fewer effects on the frontoparietal cortex in the unconscious than the conscious state. Whichever hypothesis is true, our present finding emphasizes the link between frontoparietal dynamical complexity and bottom-up interaction. It inspires a novel debate on the organization of the state-dependent cortical dynamics by suggesting that the interaction originates from the sensory cortex, which seems to be of much greater importance than previously expected.

In a current theory of consciousness, the high complexity and integrative interaction have been proposed as two important aspects of the conscious experience [4], but their relationship remains elusive. A previous study that quantified dynamical complexity [11,38] did not focus on spatial distribution of the complexity measures across brain areas, or on, its relationship to information transfer. Conversely, while previous studies have reported that functional connectivity can differentiate conscious and unconscious brain states [40,55–57], this was not linked to local dynamical characteristics. Hence, the present result establishes for the first time tight relations between these two concepts by quantifying them in a unified framework, and by revealing structural correlates of cognitive brain states. Our findings on an area-specific consciousness analogue in the frontoparietal cortex is concordant with its proposed role as a workspace for information sharing [17]. However, the present embedding-based approach conceptually advances this model by suggesting that its highly complex dynamics are the substrate of embedding the information across wide-spread brain areas, specifically in conscious states (Fig 4B). Our data and model suggest that, although the frontoparietal area is a key region, its characteristic dynamical complexity is likely to be a consequence of integrating brain-wide information including the sensory cortex. In this sense, the present results are also consistent with the view that finds the basis of consciousness in brain-wide interaction, rather than in a specific area [4,19,20]. A particularly unique aspect of the cross-embedding concept is that the information integration does not necessarily require observation of spatially distributed dynamics, but can be achieved by localized complex dynamics that embeds the global state.

More generally, the cross-embedding property highlights the universal importance of neuronal dynamics in information coding. Embedding describes the observability of information in one brain area by another area, fulfilling a prerequisite for the actor-critic models of brain systems [58]. While a similar concept has been proposed in terms of anatomical connectivity as an internal monitoring property [59], it has not been linked to neuronal dynamics. Complex dynamic in one area that embeds a high dimensional attractor is generally capable of differentiating a large number of states that characterize its entire “upstream-area”. For example, the embedding dimensions outside of the visual cortex were typically larger than one (Fig 6B), indicating that substantial information is embedded not only in single momentary states, but also in complex temporal sequences of neuronal activity. How the brain utilizes such temporally encoded information remains an open problem, nevertheless, temporal sequences in non-stationary attractor dynamics have been suggested to subserve behaviorally relevant information [60,61]. Based on the current delay-embedding framework, one can extract more in-depth information about an attractor’s topology [15,16] and its characterization would facilitate an understanding of dynamic information coding and messaging across brain areas. Together, these results suggest that computational approaches based on dynamical cross-embedding will be generally applicable to study complex neuronal interactions in large-scale physiological dynamics of the brain.

## Materials and Methods

### Ethics statement

All the experimental procedures were approved by the RIKEN Ethics Committee.

### Electrophysiological recording

We used ECoG data that were recorded from four monkeys. 128 channel ECoG signals were recorded from a wide field of a hemisphere for each monkey brain, covering the lower and higher visual cortices, temporal cortex, parietal cortex, motor and somatosensory cortex, premotor cortex, and lateral and medial prefrontal cortices; the medial prefrontal cortex was recorded from two of the four male monkeys (three *Macaca fuscata* and one *Macaca mulatta*). The details on the recording apparatus have previously been reported [62], and part of the dataset is available online (http://neurotycho.org).

### Tasks

#### Food reaching condition

A monkey sat facing an experimenter across a table in each session. The head and a hand on the same side of the ECoG array of the monkey were fixed. Another experimenter placed food (a piece of apple) either in front of the monkey or in front of the experimenter, using a dish attached to a bar to carry the food. The dish with the food came down from the top from the monkey’s point of view. The monkey grabbed the food and ate it. The food was delivered once every 10–20 s.

#### Awake and anesthetized conditions

The monkeys were sitting calm with their head and arms restrained. A ketamine-medetomidine cocktail was injected intramuscularly, or propofol was injected intravenously [42]. Each experiment consisted of three periods: (1) awake-eyes-open, where the monkeys were in a resting state with their eyes open, (2) awake-eyes-closed, where the monkeys were in a resting state with their eyes covered, (3) deep-anesthesia, where the monkeys were in a state of loss of consciousness (LOC). LOC was defined by a monkey’s loss of responsiveness to its hand being manipulated and to having its nostril/philtrum touched with a cotton swab. We observed slow wave oscillations in the neural signal [42] as an additional confirmation of LOC.

### Data analysis

For the food-reaching experiment, the data were extracted from periods where the monkeys were moving their arms. Chewing noises were eliminated from the main analysis by excluding the post-food-grabbing periods (typically 2–15 s) where the channel-averaged signal was higher than the 75th percentile of the whole data. We did not apply pre-filtering to the ECoG signals before the subsequent analyses, except for eliminating the 50±2.5 Hz component, which included line noise due to the recording apparatus. We separated the recorded signals for the anesthetization experiment into blocks corresponding to awake-eyes-open, awake-eyes-closed, and deep-anesthesia periods, and applied embedding analysis within each block. The total data length was 400 s. Data were analyzed using *random coordinate cross-embedding* (see next section for details), which we developed to reliably measure attractor dimensions. The inter-subject average was obtained by averaging the results across electrodes within individual areas of each subject and experiment, before they were averaged across subjects and experiments. The data from the medial prefrontal cortex were analyzed for the two subjects for which they had been recorded.

### Random coordinate cross-embedding

In this section, we explain the technical details of the cross-embedding algorithm while the problem formulation and the theoretical principle themselves are presented in **Results**. The algorithm was based on the convergence cross-mapping (CCM) method, which was proposed in ecosystem analysis [21] as an extension of nonlinear state-space reconstruction [22,23,63]. Although the original method was shown to characterize the causal interaction in ecological system with 2~5 variables, its applicability to analysis of dynamical complexity in large-scale and heterogeneous systems (such as the brain) has been not clear. The CCM alone does not tell us how the causal interactions are related to the emergent neural dynamics and functions. Moreover, we find that the state-space reconstruction based on the original delay-coordinate is severely affected by the heterogeneity in signal timescale across the system. Therefore, we developed a method to simultaneously quantify the causal interaction and dynamical complexity, by utilizing random projection in the reconstructed state space.

We quantified the pairwise dynamical relationships based on a delay-embedding theorem in nonlinear dynamical systems including external forces [26,64–66]. We first reconstructed the attractor manifold for each node *x* in the delay-coordinates, ${\underline{x}}_{t}^{dmax}=\;\left(x_{t},\;x_{t-\tau },\;\ldots ,\;x_{t-\left(dmax-1\right)\tau }\right)$, where *dmax* represents the maximum number of dimensions (number of delay-coordinates) to be considered, *t* is the time point, and *τ* is the unit delay length. Although the original protocol uses this delay-vector for subsequent nonlinear forecasting analysis [22,23,63], we found that it makes the results vulnerable to the time-scale heterogeneity in the system. We avoided this problem by projecting delay vector ${\underline{x}}_{t}^{dmax}$ to a randomized coordinate space by multiplying a square random matrix, ***R***, from the left to obtain a transformed vector: $x_{t}^{dmax}=\;R\;{\underline{x}}_{t}^{dmax}$, which is equivalent to considering the time series convolved with preset random filters [In our dataset, the distribution of random numbers did not have much effect on the results, at least for widely-used distributions (e.g., Gaussian or uniform distribution); in the current analysis, we used Gaussian distribution centered at zero with standard deviation of one]. A *d*-dimensional delay vector $x_{t}^{d}$ was constructed by selecting the first *d*(≤ *dmax*) components of $x_{t}^{dmax}$. The topological embeddedness for signal *y* by another signal *x* was quantified based on the correlation coefficient, $\rho_{y_{t},\hat{y}\left(x_{t}{}^{d}\right)}$, between the true (*y*_*t*_) and forecast (${\hat{y}}_{t}\left(x_{t}^{d}\right)$) signals, where

$${\hat{y}}_{t}=\underset{t^{\prime }\;s.t.\;\;x_{t\prime }^{d}\in B\left(x_{t}^{d}\right)}{\sum }w\left(\left|x_{t\prime }^{d}-x_{t}^{d}\right|\right)\;y_{t\prime }$$

with the k-nearest neighbor set $B\left(x_{t}^{d}\right)$ of $x_{t}^{d}$ in the delay-coordinate space. We set *k* = 4, weight $w\left(\left|x_{t\prime }{}^{d}-x_{t}{}^{d}\right|\right))=e^{-\left|x_{t\prime }{}^{d}-x_{t}{}^{d}\right|}/\Sigma_{x_{t\text{'}}{}^{d}\in B\left(x_{t}{}^{d}\right)}\;e^{-\left|x_{t\prime }{}^{d}-x_{t}{}^{d}\right|}$, and $\left|x_{t\text{'}}^{d}-x_{t}^{d}\right|$ as the square distance between $x_{t\text{'}}^{d}$ and $x_{t}^{d}$ in the data analysis. Note that $\rho_{y_{t},\hat{y}\left(x_{t}{}^{d}\right)}=1$ means perfect embedding, where observing $x_{t}^{d}$ completely eliminates the uncertainty of estimating state y and the conditional probability density distribution $\text{P}(y_{t}|x_{t}^{d})$ becomes a delta function. The *relative embeddedness* was derived by subtracting the embeddedness with *d* = 1 from that with *d* = d* to extract the coupling of complex temporal structures, which is not reflected in correlation.

All the analyses were conducted after normalizing the signal variance to one, in order to avoid heterogeneity in signal amplitude affecting the estimation accuracy. All estimation accuracy was measured with two-fold cross-validation, in which 1000 uniformly subsampled time points (*t*) from the latter-half of the data were predicted based on the embedding relation to the former-half, to avoid the confounding effects of over-fitting [27].

This procedure of random projection was introduced to avoid systematic biases in estimating the embedding dimension based on finite data sets. Adjacent components (e.g., *x*_*t*_, and *x*_t−τ_) typically have some non-zero correlation in a standard construction of delay-coordinate vector ${\underline{x}}_{t}^{dmax}$, depending on the signal timescale. A greater number of dimensions may be required to untangle the attractor manifold due to non-negligible correlation among the delay coordinates, leading to an overestimation of *complexity*. In addition, since the magnitude of overestimation depends on the signal timescale, the estimated *complexities* will be contaminated by variations in signal autocorrelation [27,67]. This problem does not arise after the effect of signal autocorrelation [67,68] is eliminated by applying the above-mentioned coordinate-randomization procedure. Indeed, the simulation demonstrated that estimated *complexity* typically became more accurate with the randomization procedure (Fig 2C).

The intuition behind random matrix multiplication is as follows. When the studied signal has relatively slow dynamics, the signal values at two adjacent time points, *x*(*t*) and *x*(*t*-*τ*), can be similar if unit delay *τ* is small compared to the time constant of the signal. In this case, observing *x*(*t*-*τ*) in addition to *x*(*t*) does not practically add much information about the system state. As a consequence, small *τ* can lead to overestimation of dimensionality in standard delay coordinates. Hence, estimated dimension sensitively depends on the choice of *τ* in pre-randomized delay coordinates. Using the randomized delay coordinates alleviates this problem. In this case, each observation is linear sum of observations at across widely different time delays, where a weighted sum of observations at intermediate delays always well characterizes the system dynamics. As we explained above, summed observations at small delays do not add much information but are not harmful. Summed observations at large delays can add variability if the system is very noisy (because noise can accumulate in time) but generally does not bias characterization of system’s state. Hence, the choice of the maximal delay requires much less tuning than the choice of *τ* in the standard delay coordinates.

### Complexity

The *complexity* of dynamics concerning pairwise interaction was quantified using minimum embedding dimension d* that yielded ≥ 95% of optimal estimation accuracy ${\text{max}}_{d}\;\;\rho_{y,\hat{y}\left(x_{t}{}^{d}\right)}$, which is an extension of the false-neighbor method [27,28,68] to the present cross-embedding. (Our ECoG results were robust when *complexity* was instead defined as the minimum embedding dimension that yielded 90% or 100% of the optimal estimation accuracy.) Note that in real data with a limited sample size, the accuracies sometimes did not have plateaus but have a peak followed by a gradual decrease [23]. In this case, *complexity* corresponds approximately to a dimension that maximizes estimation accuracy. Estimating from the upstream to downstream nodes is generically incomplete (correlation coefficient < 1) because the latter does not embed the former; even in such cases, estimation accuracy depends on the embedding dimension, and we can quantify *complexity* with the same procedure. While the method cannot estimate the attractor dimension of the target electrode if the cross-embedding method detects no interactions, this only occasionally happened in our analyses of the ECoG data. As expected from this characteristic, our results were qualitatively and quantitatively the same when we limited our analyses only to most strongly interacting pairs of electrodes.

### Directionality

The *directionality* in interaction from *x* to *y* was quantified by the difference between optimal estimation accuracies: $\rho_{y,\hat{y}\left(x_{t}{}^{d}\right)}-\rho_{x,\hat{x}\left(y_{t}{}^{d}\right)}$. This takes a positive value when there is directed interaction from x to *y*. Note that stochastic components within an upstream system is not embedded by downstream dynamics since the embedding requires deterministic nature of dynamics. However, the directionality of causality between as pair of nodes should be detected by a node asking the other node whether there is any asymmetry of observability (conditional entropy) of a node by the other node [21], even under some stochasticity due to system noise (S4 Fig). It is worth noting that detecting a clear causal relationship by Transfer entropy [69,70], Granger causality [71] or spectral Granger causality [72,73] is not straightforward unless the system’s dynamical properties are well known, due to the confounding effects of phase delay [74] or self-predictability in deterministic dynamics [21].

### Parameter selection

In the present analysis, we used unit delay *τ* = 20 ms and embedding dimension *dmax* = 30 in the main analysis. The optimal unit-delay depends on noise/dynamical property, and again, has to be selected generally depending on each dataset to be studied. Note that our point in Fig 7 is not to show that 20-ms delay is “the optimal” but to demonstrate that the complexity and relative relationships in terms of network upstream-downstream is robust to selection of unit delay. The embedding dimension, *dmax*, does not affect the results in theory, as long as our methodological assumptions hold and if we set *dmax* sufficiently larger than true dimensions of attractor. In practice in the presence of noise in the system, however, the embedding performance slowly falls if we choose too large *dmax*. However, this *dmax* dependency is not very sensitive because intermediate delays always provide good signal about system’s state and, while large delays gradually add some variability, they do not bias the estimate. Since we do not know a-priori the maximum dimensionality of attractors in general, *dmax* has to be selected ad hoc manner depending on data to be studied. In this study, we set *dmax* = 30 empirically by observing that the accuracy of nearest-neighbor mapping (measure of “embeddedness”) saturates, at most, around dimensions 10.

In the current data, we used total data points in each segment at order of 10000 to obtain robust results. When we decrease the number of data points (e.g., 6000 or 2000), the accuracy of nearest-neighbor mapping were degraded, although the relative patterns (e.g., asymmetry in mapping accuracy) tended to be preserved. Regarding how to estimate the sufficient data length, one rough estimate is based on the dimension of an attractor, *d*. For example, if *n* data points per dimension are needed for accurate prediction, the total points required would be ~*n* ^*d*^. While the number of required data points also depends on spatial roughness of the attractor, this provides a reasonable estimate. A more sophisticated estimate may be possible by fitting the attracter using a manifold learning algorithm.

### Effect of data variance

Although we normalized the data before the present analyses to make the results comparable to the correlation analysis and to rule out the possibility that the heterogeneity in signal amplitude variability affected the results, the embedding analysis is, in principle, scale invariant. The mathematical concept of embedding relies only on the topological property of attractor dynamics, which has by definition nothing to do with the scale of the signal amplitude (e.g., variance). In this sense, the principle of method is scale invariant. In practical implementation, however, the result of the analysis can be affected by the scale of signal under a limited data length. For a concrete example, we used a nearest neighbor model to construct a mapping from a reconstructed attractor to the other one. In this case, the results depend on how we define the “neighbor” of a data point. Unless we have infinitely long data, the data points are distributed on the attractor with some sparcity. Keeping the number of total data points fixed, the larger signal scale leads to the smaller absolute density of data points (number of data points in a unit cube/sphere in the state space). If the neighbor of a data point were defined with the unit cube/sphere around it, the number of other data points inside the neighbor would be affected by the absolute density of data points, and thus the result would depend on the scale of signal. On the other hand, in the present method, we defined the neighbor not by unit cube/sphere but by the simplex spanned by *k*-nearest points of each data point with keeping *k* constant. In this method, the number of the data points to construct nearest-neighbor mapping does not depend on the scale. We also confirmed that the normalization procedure was not critical in the present finding (S1E Fig).

### Other complexity measures

The correlation dimension was computed with the algorithm proposed by [29]; the PCA dimension was quantified by the number of dimension explaining 90% of the all variance; the permutation entropy was derived using the algorithm proposed by [31]; the algorithmic complexity was derived with the method proposed by [75]; the complexity with standard delay-coordinate was given as the optimal embedding dimension in the algorithm proposed by [21].

### Artificial system

The example dynamics in Fig 1C–1F were generated based on the following coupled Rössler oscillators:

$$T{\dot{\xi }}_{1}=-\left(\eta_{1}+\zeta_{1}\right)\;(\xi_{2}+11)/2,\;\;T{\dot{\xi }}_{2}=-\left(\eta_{2}+\zeta_{2}\right),$$

$$T{\dot{\eta }}_{1}=\xi_{1}+0.2\eta_{1},\;\;T{\dot{\eta }}_{2}=\xi_{2}+0.22\eta_{2},$$

$$T{\dot{\zeta }}_{1}=\zeta_{1}\left(\xi_{1}-5.7\right)+0.2,\;\;T{\dot{\zeta }}_{2}=\zeta_{1}\left(\xi_{1}-5.6\right)+0.2,$$

where the dots indicate temporal differentiation; $\vec{x}$ *= (ξ*_1_, *η*_1_, *ζ*_1_), $\vec{y}$ = (*ξ*_2_, *η*_2_, *ζ*_2_), and (*x*_obs_, *y*_obs_) = (*ξ*_1_, *ξ*_2_) in Fig 1C. Parameter *T* controls the timescale of the dynamics. The signals were generated using a fourth-order Runge-Kutta method with a time step of 0.01, and subsampled with a time step of 0.1 in the embedding analysis, where we used *τ* = 4 and *dmax* = 20.

### Network simulation

In Fig 8, the brain dynamics were simulated with the following difference equations:

$$x_{i}\left(t\right)=f\left(\underset{j}{\sum^{\text{​}}}J_{ij}x_{j}\left(t-1\right)\right)+b_{i}\left(t\right),$$

Where *x*_*i*_(*t*) is activity of *i*th node at time step *t*, *J*_ij_ is the connectivity from *j*th to *i*th node, and *f*(*x*) = *x* exp[3(1 –*x*)]. The activation function, *f*, is interpreted as a qualitative model of balanced local circuit, although our aim here is not to provide a detailed model of nervous systems but to demonstrate the basic mechanisms with a simplified example. We set *J*_*ii*_ = 1 for all *i*. Inter-node connectivity (*J*_*ii*_) was randomly sampled from a normal distribution [mean = 0, and standard deviation = 0.1/*N* (within cluster) or 0.5/*N* (between cluster), where *N* is the total number of nodes in the whole network], or set as zero, depending on the models; in the model 3, the connectivity within the frontoparietal cluster was doubled, except for the self-feedback (*J*_*ii*_). *b*_*i*_(*t*) is the bias input; to add the baseline correlation, we assumed the bias term irrelevant to brain state, and modeled it with a sinusoidal function with random amplitudes and phase. We ran the simulation for 1000 time steps, with 5 nodes for each clusters for illustrative purpose (increasing the number of node had not much effect on the simulation results; note also that the number of nodes does not directly reflect the number of ECoG electrodes). After the generating simulated dynamics, we performed the same cross-embedding analysis as the one used in the main ECoG results (except for that here we used the unit time-delay of 1 time step).

## Supporting Information

S1 Fig Robustness to analytical methods. Trends are reproducible across recording days, animals, and types of anesthetic chemicals. (A) Bipolar analysis, in which differential signals from neighboring electrode pairs were used as data. (B) Analysis without eliminating chewing period (see Materials and Methods). (C) The results in which the random matrix with orthogonal basis was used for the coordinate transformation. (D) Results using electrode pairs that showed significant interaction (64.5 ± 3.24% [mean ± SEM] of pairs that have P < 0.01 by the Pearson correlation test for the embedding-based forecasting performance). (E) Results without normalization of signal variability before the analysis. (Top row) Convention follows that in Fig 5A. (Bottom row) Convention follows that in Fig 5B.
(PDF)

S2 Fig Multi-area dynamics have complex structures. Dynamics of the all electrodes shown in three principal components, where the 128 electrode dynamics were reduced using PCA, based on the correlation matrices. Results for (left) awake-eyes-closed and (right) propofol anesthesia in a single subject are shown.
(PDF)

S3 Fig Distribution of interaction strength. (A) The distribution of p-value in the embedding-based forecasting performance (correlation between forecasted and true data). The majority (64.5%) of electrode pairs showed significant interaction (p<0.05). (B) The p-value distribution in transfer entropy, where we used nonuniform embedding-dimension technique [70] for a fair comparison. In panels A and B, data from the all experimental sessions are combined. Filled bar: significant pairs; open bars: not significant pairs.
(PDF)

S4 Fig Robustness of embedding results under system noise. Results of the cross-embedding analysis where moderate system noise were added to the system shown in Fig 1C–1F. Gaussian random noise were added to each node in every time step. The noise level denotes the standard deviation of noise component per unit time, where the dynamic range of signal is about ±1. (System noise > 0.05 per every unit-time made the simulated attractor dynamics unstable) (A) The estimated *complexity*. (B) The estimated directionality. Note that the polarity of directionality is maintained when the noise level vary.
(PDF)

S5 Fig Robustness of correlation analyses to removal of short-range interactions. (A and B) The same as Fig 3M and 3N, but the electrode pairs within the same cortical areas were eliminated from the analyses.
(PDF)
