# Supplementary figures and images for: Untangling Brain-Wide Dynamics in Consciousness by Cross-Embedding
Source: PLoS Comput Biol. 2015 Nov 19;11(11):e1004537. doi: 10.1371/journal.pcbi.1004537 (PMC4652869; doi:10.1371/journal.pcbi.1004537)

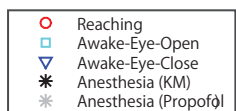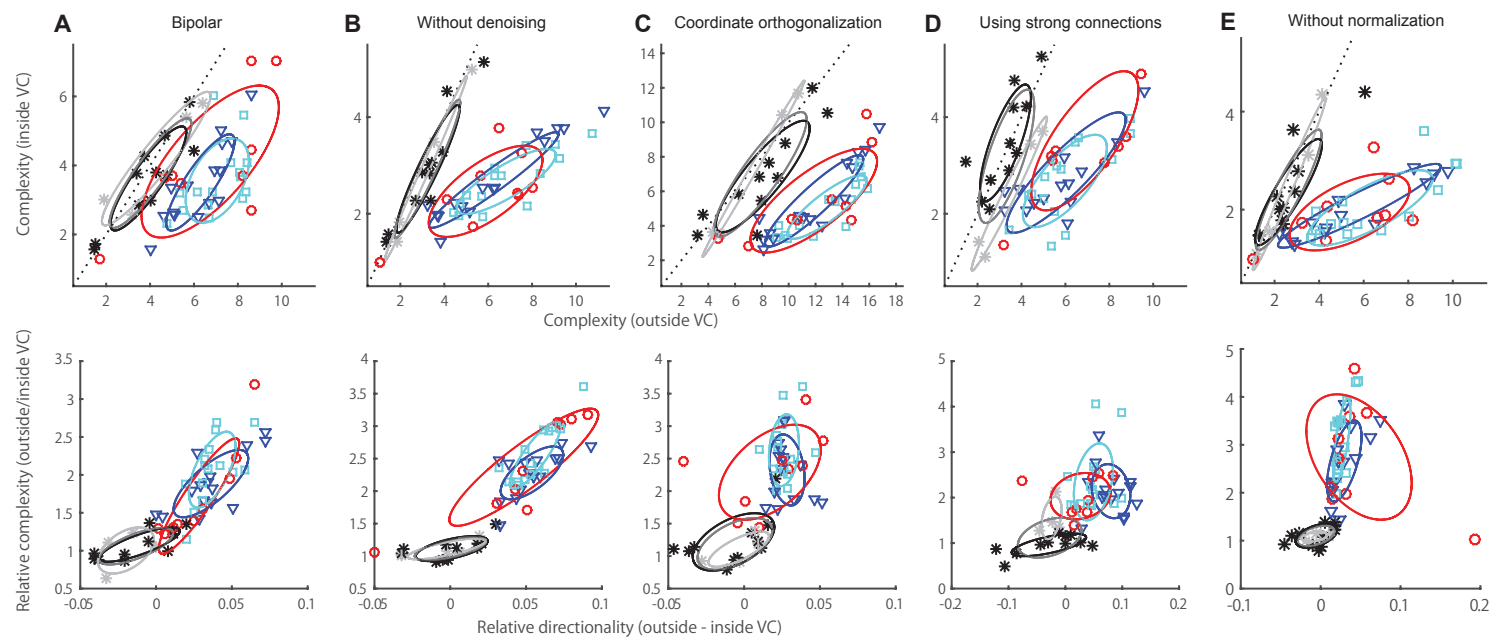

Supplement: S1 Fig — Trends are reproducible across recording days, animals, and types of anesthetic chemicals. (A) Bipolar analysis, in which differential signals from neighboring electrode pairs were used as data. (B) Analysis without eliminating chewing period (see Materials and Methods). (C) The results in which the random matrix with orthogonal basis was used for the coordinate transformation. (D) Results using electrode pairs that showed significant interaction (64.5 ± 3.24% [mean ± SEM] of pairs that have P < 0.01 by the Pearson correlation test for the embedding-based forecasting performance). (E) Results without normalization of signal variability before the analysis. (Top row) Convention follows that in Fig 5A. (Bottom row) Convention follows that in Fig 5B. (PDF) [file pcbi.1004537.s001.pdf]

Awake (eyes closed)

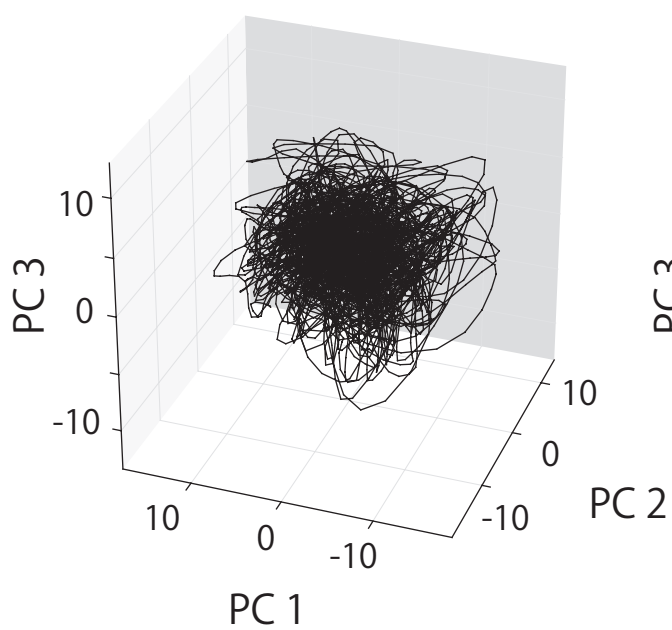

Anesthetized (propofol)

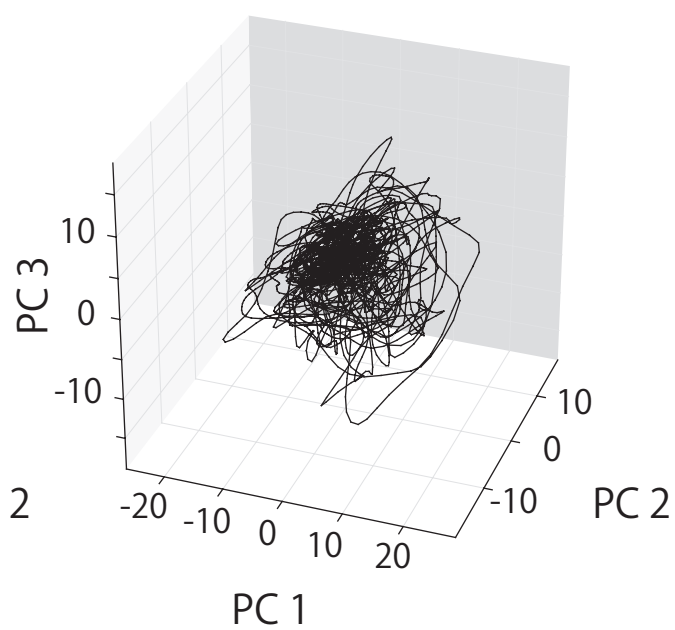

Supplement: S2 Fig — Dynamics of the all electrodes shown in three principal components, where the 128 electrode dynamics were reduced using PCA, based on the correlation matrices. Results for (left) awake-eyes-closed and (right) propofol anesthesia in a single subject are shown. (PDF) [file pcbi.1004537.s002.pdf]

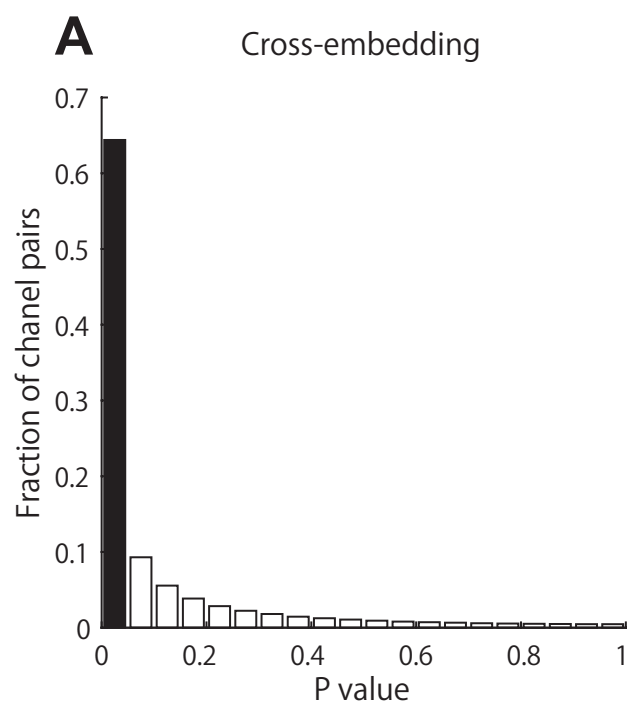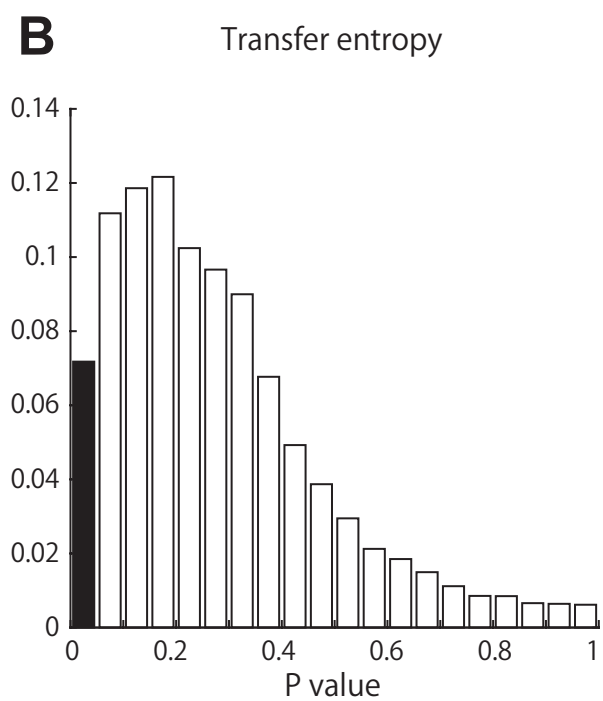

Supplement: S3 Fig — (A) The distribution of p-value in the embedding-based forecasting performance (correlation between forecasted and true data). The majority (64.5%) of electrode pairs showed significant interaction (p<0.05). (B) The p-value distribution in transfer entropy, where we used nonuniform embedding-dimension technique [70] for a fair comparison. In panels A and B, data from the all experimental sessions are combined. Filled bar: significant pairs; open bars: not significant pairs. (PDF) [file pcbi.1004537.s003.pdf]

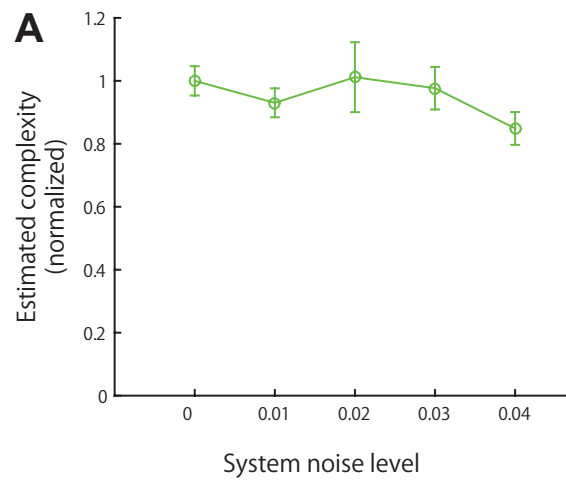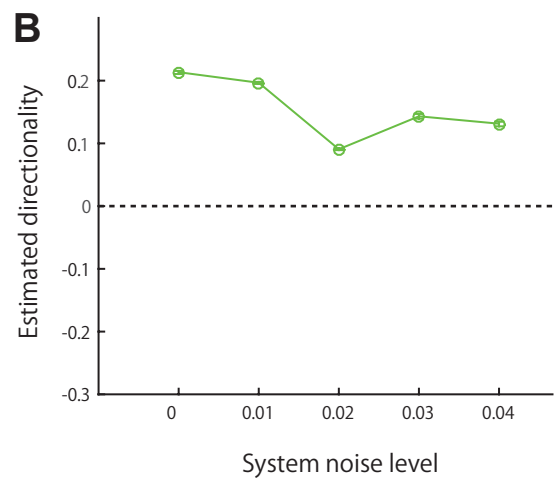

Supplement: S4 Fig — Results of the cross-embedding analysis where moderate system noise were added to the system shown in Fig 1C–1F. Gaussian random noise were added to each node in every time step. The noise level denotes the standard deviation of noise component per unit time, where the dynamic range of signal is about ±1. (System noise > 0.05 per every unit-time made the simulated attractor dynamics unstable) (A) The estimated complexity. (B) The estimated directionality. Note that the polarity of directionality is maintained when the noise level vary. (PDF) [file pcbi.1004537.s004.pdf]

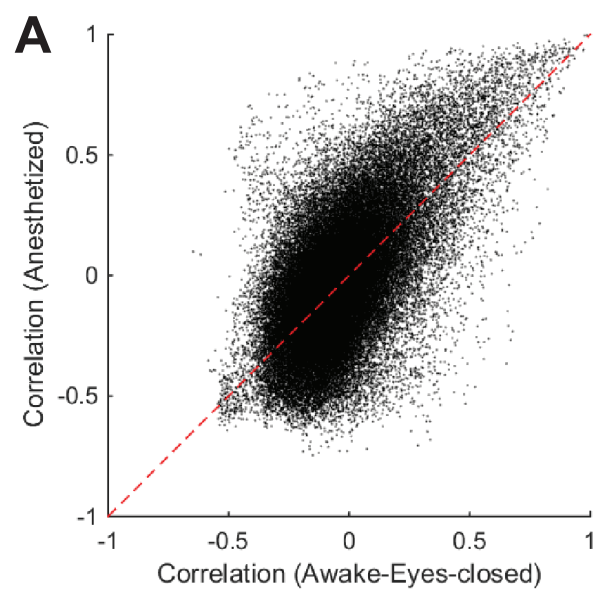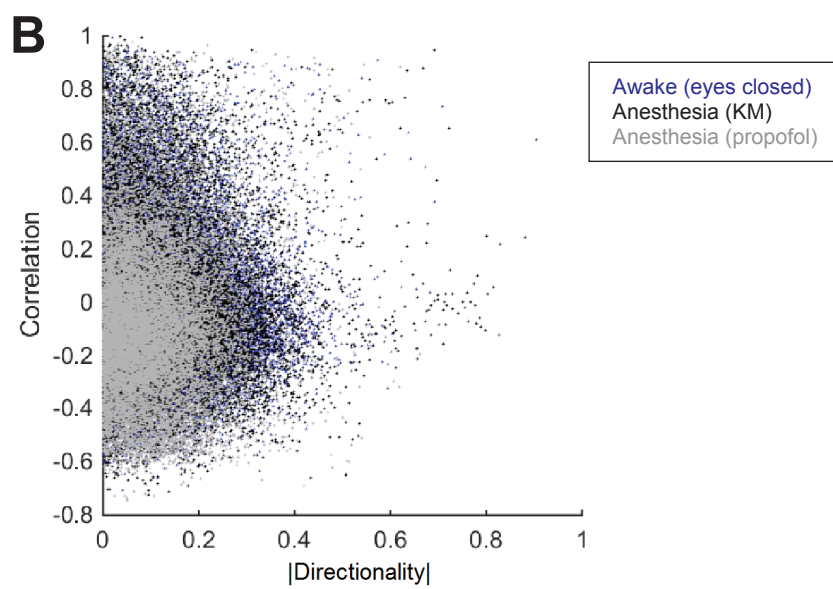

Supplement: S5 Fig — (A and B) The same as Fig 3M and 3N, but the electrode pairs within the same cortical areas were eliminated from the analyses. (PDF) [file pcbi.1004537.s005.pdf]
